# Supplementary material for: Simultaneous application of enzyme and thermodynamic constraints to metabolic models using an updated Python implementation of GECKO
Source: Microbiol Spectr. 2023 Oct 16;11(6):e01705-23. doi: 10.1128/spectrum.01705-23 (PMC10783817; doi:10.1128/spectrum.01705-23)
Supplement: S2 file — Jupyter notebook to reproduce figures 1, 2, 3. [file spectrum.01705-23-s0002.html]

proteomics\_data\_relaxations\_try\_work


# Proteomics EC GEM integration¶

#### Table of contents¶

- Data preparation
  - Uniprot Mapping
  - Protein unit conversion
- Application to model
- Relaxation runs
  - Relaxation Aggregated by biological replicates
  - Sensibility Aggregated by biological replicates
  - Get average enzyme saturation

In [1]:

```
import re
import warnings
from collections import Counter
from copy import deepcopy
from functools import reduce
from math import isnan, sqrt
from pathlib import Path

import geckopy
import numpy as np
import pandas as pd
import plotly.express as px
from geckopy.experimental import from_mmol_gDW
from geckopy.experimental.molecular_weights import extract_proteins
from geckopy.experimental.relaxation import (
    Objective_rule,
    elastic_upper_relaxation,
    get_upper_relaxation,
)
from tqdm import tqdm
```

## Data preparation¶

### Uniprot extraction¶

In [2]:

```
DATA = Path.cwd().parent / "data"
ticktext = [
    "Reference EQ353",
    "C-limitation (400 µM 3MBA) NQ1243",
    "R-limitation NCM3722",
    "C-limitation (100 µM 3MBA) NQ1243",
    "C-limitation NQ1243",
    "C-limitation (400 µM 3MBA) NQ1390",
    "C-limitation (40 µM 3MBA) NQ1390",
    "A-limitation (20 µM IPTG) NQ393",
    "A-limitation (30 µM IPTG) NQ393",
    "A-limitation (40 µM IPTG) NQ393",
    "A-limitation (50 µM IPTG) NQ393",
    "A-limitation (100 µM IPTG) NQ393",
    "C-limitation (400 µg 3MBA) NQ1390",
    "R-limitation (2µg CAF) NCM3722",
    "R-limitation (4µg CAF) NCM3722",
    "R-limitation (6µg CAF) NCM3722",
    "R-limitation (8µg CAF) NCM3722",
]
tickvals = [
    "['A1-1', 'A1-2', 'A1-3', 'C1', 'F1-1', 'F1-2', 'F1-3']",
    "['C3', 'F4']",
    "['C2', 'A2', 'H1', 'H5']",
    "['C4', 'C8', 'F5']",
    "['C5', 'D6', 'F6']",
    "['C6', 'F7']",
    "['C7', 'D7', 'F8']",
    "['D1']",
    "['D2']",
    "['D3']",
    "['D4']",
    "['D5', 'F2', 'F3']",
    "['D8']",
    "['E1']",
    "['E2']",
    "['E3']",
    "['E4']",
]
```

In [3]:

```
ev9 = pd.read_excel(
    DATA / "msb20209536-sup-0010-datasetev9.xlsx",
    engine="openpyxl",
    sheet_name=1,
)
```

In [4]:

```
print(
    pd.read_excel(
        DATA / "msb20209536-sup-0010-datasetev9.xlsx", engine="openpyxl", sheet_name=0
    ).iloc[1, 0]
)
```

```
Description
```

In [5]:

```
ev9.describe()
```

Out[5]:

|  | A1-1 | A1-2 | A1-3 | C1 | F1-1 | F1-2 | F1-3 | C2 | C3 | C4 | ... | D5 | F2 | F3 | A2 | E1 | E2 | E3 | E4 | H1 | H5 |
| --- | --- | --- | --- | --- | --- | --- | --- | --- | --- | --- | --- | --- | --- | --- | --- | --- | --- | --- | --- | --- | --- |
| count | 4342.000000 | 4342.000000 | 4342.000000 | 4342.000000 | 4342.000000 | 4342.000000 | 4342.000000 | 4342.000000 | 4342.000000 | 4342.000000 | ... | 4342.000000 | 4342.000000 | 4342.000000 | 4342.000000 | 4342.000000 | 4342.000000 | 4342.000000 | 4342.000000 | 4342.000000 | 4342.000000 |
| mean | 0.000230 | 0.000230 | 0.000230 | 0.000230 | 0.000230 | 0.000230 | 0.000230 | 0.000230 | 0.000230 | 0.000230 | ... | 0.000230 | 0.000230 | 0.000230 | 0.000230 | 0.000230 | 0.000230 | 0.000230 | 0.000230 | 0.000230 | 0.000230 |
| std | 0.001224 | 0.001230 | 0.001247 | 0.001244 | 0.001233 | 0.001254 | 0.001281 | 0.001355 | 0.001351 | 0.001288 | ... | 0.001388 | 0.001369 | 0.001353 | 0.001224 | 0.001153 | 0.001132 | 0.001131 | 0.001150 | 0.001205 | 0.001168 |
| min | 0.000000 | 0.000000 | 0.000000 | 0.000000 | 0.000000 | 0.000000 | 0.000000 | 0.000000 | 0.000000 | 0.000000 | ... | 0.000000 | 0.000000 | 0.000000 | 0.000000 | 0.000000 | 0.000000 | 0.000000 | 0.000000 | 0.000000 | 0.000000 |
| 25% | 0.000000 | 0.000000 | 0.000000 | 0.000000 | 0.000000 | 0.000000 | 0.000000 | 0.000000 | 0.000000 | 0.000000 | ... | 0.000000 | 0.000000 | 0.000000 | 0.000000 | 0.000000 | 0.000000 | 0.000000 | 0.000000 | 0.000000 | 0.000000 |
| 50% | 0.000000 | 0.000000 | 0.000000 | 0.000000 | 0.000000 | 0.000000 | 0.000000 | 0.000000 | 0.000000 | 0.000000 | ... | 0.000000 | 0.000000 | 0.000000 | 0.000000 | 0.000000 | 0.000000 | 0.000000 | 0.000000 | 0.000000 | 0.000000 |
| 75% | 0.000084 | 0.000083 | 0.000081 | 0.000088 | 0.000088 | 0.000086 | 0.000086 | 0.000077 | 0.000078 | 0.000082 | ... | 0.000078 | 0.000076 | 0.000076 | 0.000084 | 0.000086 | 0.000087 | 0.000085 | 0.000085 | 0.000085 | 0.000084 |
| max | 0.039933 | 0.040456 | 0.042874 | 0.039796 | 0.039674 | 0.041150 | 0.044211 | 0.059822 | 0.057926 | 0.049607 | ... | 0.058102 | 0.062145 | 0.057935 | 0.049734 | 0.038487 | 0.034953 | 0.030230 | 0.027281 | 0.046204 | 0.044436 |

8 rows × 36 columns

In [6]:

```
PROT_PATTERN = re.compile(
    r"[OPQ][0-9][A-Z0-9]{3}[0-9]|[A-NR-Z][0-9]([A-Z][A-Z0-9]{2}[0-9]){1,2}"
)
```

In [7]:

```
ev9["Protein ID"].isna().sum() / ev9.shape[0]
```

Out[7]:

```
0.027637033625057577
```

2.7% of proteins do not have an identifier. We can just remove them for now.

In [8]:

```
ev9_valid = ev9[~ev9["Protein ID"].isna()]
```

In [9]:

```
ev9_valid["Protein ID"].str.match(PROT_PATTERN).all()
```

Out[9]:

```
True
```

All proteins seems to match a Uniprot ID.

In preparation for the unit conversions, extract the molecular weights querying uniprot.

In [10]:

```
prot_list = ev9_valid["Protein ID"].to_list()
prot_reactions = [f"prot_{prot}" for prot in prot_list]
prot_dict = dict(zip(prot_list, prot_reactions))
```

In [11]:

```
ev9_valid["MW"] = extract_proteins(None, prot_dict)["MW"]
```

```
100%|█████████████████████████████████████████████████████████████████████████████████████████████████████████████████████████████████████████████████████████| 3/3 [00:45<00:00, 15.07s/it]
/tmp/ipykernel_60974/1682032849.py:1: SettingWithCopyWarning: 
A value is trying to be set on a copy of a slice from a DataFrame.
Try using .loc[row_indexer,col_indexer] = value instead

See the caveats in the documentation: https://pandas.pydata.org/pandas-docs/stable/user_guide/indexing.html#returning-a-view-versus-a-copy
  ev9_valid["MW"] = extract_proteins(None, prot_dict)["MW"]
```

In [12]:

```
ev9_valid = ev9_valid[~ev9_valid["MW"].isna()]
ev9_valid.to_csv(DATA / "EV9_clean_MW.tsv", sep="\t")
```

### Protein unit conversion¶

In [13]:

```
ev9_valid = pd.read_csv(DATA / "EV9_clean_MW.tsv", sep="\t")
```

In [14]:

```
ev9_valid = ev9_valid.loc[:, ~ev9_valid.columns.str.startswith("Unnamed")]
```

Let's put it in tidy format to make the unit conversions.

In [15]:

```
ev9_tidy = ev9_valid.melt(
    ["MW", "Protein ID", "Gene name", "Gene locus"],
    var_name="Sample",
    value_name="Mass_fraction",
)
```

In [16]:

```
g_per_gDW = 0.448
```

\begin{align}
\frac{\mu g}{\mu m^3}\_k &= \phi\_k 13.5 x 10^{-8} \\
v\_{ODmL} &= \frac{X \mu m^3}{OD mL} \\
\frac{mmol}{g\_{DW}}\_k &= \frac{k \mu g}{\mu m^3}\_k \frac{1 g}{10^6 \mu g} \frac{1000 mmol\_k}{M\_{w,k} g} v\_{ODmL} \frac{OD mL}{320 \mu g protein} \frac{10^6 \mu g \text{protein}}{g \text{protein}} \frac{0.448g protein}{g\_{DW}}
\end{align}

In [17]:

```
growth_conditions = pd.read_excel(
    DATA / "msb20209536-sup-0004-datasetev3.xlsx", sheet_name=1, engine="openpyxl"
)
```

In [18]:

```
sample_growth = growth_conditions[["Sample ID", "Growth rate (1/h)"]].set_index(
    "Sample ID"
)["Growth rate (1/h)"]
```

In [19]:

```
ev9_v_ODmL = ev9_tidy.Sample.apply(lambda x: sample_growth.loc[x] * -0.3e9 + 2.83e9)
```

In [20]:

```
ev9_tidy["mmolgDW"] = (
    13.5e-8 * ev9_tidy.Mass_fraction * 1000 / ev9_tidy.MW * ev9_v_ODmL / 320 * g_per_gDW
)
```

In [21]:

```
ev9_tidy.mean()
```

```
/tmp/ipykernel_60974/3197857229.py:1: FutureWarning: The default value of numeric_only in DataFrame.mean is deprecated. In a future version, it will default to False. In addition, specifying 'numeric_only=None' is deprecated. Select only valid columns or specify the value of numeric_only to silence this warning.
  ev9_tidy.mean()
```

Out[21]:

```
MW               34992.280712
Mass_fraction        0.000242
mmolgDW              0.000010
dtype: float64
```

In [22]:

```
ev9_tidy[["Protein ID", "mmolgDW", "MW", "Sample"]].to_csv(
    DATA / "EV9_final.tsv", sep="\t", index=False
)
```

In [23]:

```
ev9_final = pd.read_csv(DATA / "EV9_final.tsv", sep="\t")
```

In [24]:

```
ev9_final = ev9_final[~ev9_final.mmolgDW.isna()]
```

In [25]:

```
df_prot = ev9_final
```

In [26]:

```
df_prot.to_csv(DATA / "processed_proteins.tsv", sep="\t", index=False)
```

### Application to model¶

The absolute proteomics will be applied to the model for every condition.

In [27]:

```
df_prot = pd.read_csv(DATA / "processed_proteins.tsv", sep="\t")
```

In [28]:

```
df_prot.iloc[[0, 1, 2, 3, -5, -4, -3, -2, -1], :]
```

Out[28]:

|  | Protein ID | mmolgDW | MW | Sample |
| --- | --- | --- | --- | --- |
| 0 | P46482 | 0.0 | 34775.601615 | A1-1 |
| 1 | P46481 | 0.0 | 73593.694590 | A1-1 |
| 2 | P67662 | 0.0 | 34517.064180 | A1-1 |
| 3 | P46478 | 0.0 | 7846.801510 | A1-1 |
| 147991 | P76612 | 0.0 | 48396.048400 | H5 |
| 147992 | P76613 | 0.0 | 51033.377940 | H5 |
| 147993 | P64432 | 0.0 | 26485.692260 | H5 |
| 147994 | Q46953 | 0.0 | 19254.789250 | H5 |
| 147995 | P58033 | 0.0 | 55706.098450 | H5 |

In [29]:

```
model = geckopy.io.read_sbml_ec_model(DATA / "eciML1515.xml.gz")
```

In [30]:

```
model.solver = "cplex"
```

In [31]:

```
growth_conditions = pd.read_excel(
    DATA / "msb20209536-sup-0004-datasetev3.xlsx", sheet_name=1, engine="openpyxl"
)
```

In [32]:

```
growth_conditions.head()
```

Out[32]:

|  | Sample ID | Group | Growth rate (1/h) | Strain | Growth medium | Carbon source | Nitrogen source | Supplement | Description | SWATH file name |
| --- | --- | --- | --- | --- | --- | --- | --- | --- | --- | --- |
| 0 | A1-1 | EQ353 calibration samples | 0.69 | EQ353 | MOPS (Neidhardt) | 0.2% glucose | 9.5 mM NH4Cl | NaN | Same strain and growth condition as Li et al. ... | CHLUDWIG\_K151202\_010\_SW |
| 1 | A1-2 | NaN | 0.69 | EQ353 | MOPS (Neidhardt) | 0.2% glucose | 9.5 mM NH4Cl | NaN | Same strain and growth condition as Li et al. ... | CHLUDWIG\_K151202\_084\_SW |
| 2 | A1-3 | NaN | 0.69 | EQ353 | MOPS (Neidhardt) | 0.2% glucose | 9.5 mM NH4Cl | NaN | Same strain and growth condition as Li et al. ... | CHLUDWIG\_K151202\_085\_SW |
| 3 | C1 | NaN | 0.73 | EQ353 | MOPS (Neidhardt) | 0.2% glucose | 9.5 mM NH4Cl | NaN | Biological replicate of A1 | CHLUDWIG\_K151202\_052\_SW |
| 4 | F1-1 | NaN | 0.71 | EQ353 | MOPS (Neidhardt) | 0.2% glucose | 9.5 mM NH4Cl | NaN | Biological replicate of A1 and C1 (technical r... | CHLUDWIG\_K151202\_031\_SW |

Need the growth rate $\frac{1}{h}$ (in one of the sheets).

In [33]:

```
growth_conditions.columns
```

Out[33]:

```
Index(['Sample ID', 'Group', 'Growth rate (1/h)', 'Strain', 'Growth medium',
       'Carbon source', 'Nitrogen source', 'Supplement', 'Description',
       'SWATH file name'],
      dtype='object')
```

In [34]:

```
growth_conditions.loc[growth_conditions["Sample ID"] == "A1-1", "Growth rate (1/h)"][0]
```

Out[34]:

```
0.69
```

In [35]:

```
objective_id = "BIOMASS_Ec_iML1515_core_75p37M"
```

In [36]:

```
iis_lp_counter = dict()
iis_milp_counter = dict()
```

In [37]:

```
growth_conditions["Sample ID"]
```

Out[37]:

```
0     A1-1
1     A1-2
2     A1-3
3       C1
4     F1-1
5     F1-2
6     F1-3
7       C2
8       C3
9       C4
10      C5
11      C6
12      C7
13      C8
14      D6
15      D7
16      D8
17      F4
18      F5
19      F6
20      F7
21      F8
22      D1
23      D2
24      D3
25      D4
26      D5
27      F2
28      F3
29      A2
30      H1
31      H5
32      E1
33      E2
34      E3
35      E4
Name: Sample ID, dtype: object
```

In [38]:

```
growth_conditions.loc[
    growth_conditions["Sample ID"] == "A1-3", "Growth rate (1/h)"
].iloc[0]
```

Out[38]:

```
0.69
```

# Relaxation runs¶

## Relaxation Aggregated by biological replicates¶

Lastly, do it properly with the biological replicates (but different original datasets).

#### About strains¶

EQ353 is equivalent to the model:

- Knock down in pyrE expression (corresponds to ORPTNo1, prot\_P0A7E3, which should be accounted in the proteomics data).
- Knock out of ilvG, which is already removed in the model.

NCM3722 is the model (K12 usual strain).

- Is $\Delta$ilvG also in the original K12?

NQ1243, NQ1390 (C-limitation) both have the titration construct Pu-ptsG that regulates Glc uptake externally using 3MBA (no difference is specified between those, no strains with those names are in the specified references).

- Can we expect this from proteomics?

NQ393 (A-limitation)

- Deletion of $\Delta$gdhA, GDH: H2O + L-glutamate + NADP+ = 2-oxoglutarate + H+ + NADPH + NH4+\*.
- Promoter replacement of gltBD to control (via IPTG) GOGAT expression: Gln + akg -> 2 Glu (should be accounted for in proteomics).

#### About the growth conditions¶

C-limitation (carbon)

- 3MBA supplement, which is proportional to ptsG expression (Glc uptake).

A-limitation (ammonia)

- IPTG supplement, which is proportional to GOGAT expression.

R-limitation (ribosome)

- Cloramphenicol supplements, which inhibit ribosomal translation (it is seen in a cap of the growth rate).

In [40]:

```
# so that groupby makes sense
growth_conditions.Supplement = growth_conditions.Supplement.apply(lambda x: "No" if pd.isna(x) else x)
```

In [41]:

```
(
    growth_conditions.groupby(["Strain", "Supplement"]).agg(
        {
            "Growth rate (1/h)": lambda x: x.mean() if len(x) > 1 else x.iloc[0],
            "Sample ID": lambda x: x.to_list(),
        }
    )
)
```

Out[41]:

|  |  | Growth rate (1/h) | Sample ID |
| --- | --- | --- | --- |
| Strain | Supplement |  |  |
| EQ353 | No | 0.704286 | [A1-1, A1-2, A1-3, C1, F1-1, F1-2, F1-3] |
| NCM3722 | 2 µg chloramphenicol | 0.790000 | [E1] |
|  | 4 µg chloramphenicol | 0.610000 | [E2] |
|  | 6 µg chloramphenicol | 0.490000 | [E3] |
|  | 8 µg chloramphenicol | 0.360000 | [E4] |
|  | No | 0.960000 | [C2, A2, H1, H5] |
| NQ1243 | 100 µM 3MBA | 0.706667 | [C4, C8, F5] |
|  | 400 µM 3MBA | 0.790000 | [C3, F4] |
|  | No | 0.556667 | [C5, D6, F6] |
| NQ1390 | 40 µM 3MBA | 0.340000 | [C7, D7, F8] |
|  | 400 µM 3MBA | 0.490000 | [C6, F7] |
|  | 400 µg 3MBA | 0.490000 | [D8] |
| NQ393 | 100 µM IPTG | 0.880000 | [D5, F2, F3] |
|  | 20 µM IPTG | 0.220000 | [D1] |
|  | 30 µM IPTG | 0.360000 | [D2] |
|  | 40 µM IPTG | 0.540000 | [D3] |
|  | 50 µM IPTG | 0.670000 | [D4] |

In [42]:

```
df_growth_agg_changes = growth_conditions.groupby(["Strain", "Supplement"]).agg(
    growth_mean=pd.NamedAgg(column="Growth rate (1/h)", aggfunc="mean"),
    growth_std=pd.NamedAgg(column="Growth rate (1/h)", aggfunc="std"),
    sample=pd.NamedAgg(column="Sample ID", aggfunc=lambda x: x.to_list()),
)
```

In [43]:

```
iis_lp_dict_agg = {}
iis_milp_dict_agg = {}
iis_lp_dict_agg_first = {}
iis_milp_dict_agg_first = {}
```

In [44]:

```
df_growth_agg_changes
```

Out[44]:

|  |  | growth\_mean | growth\_std | sample |
| --- | --- | --- | --- | --- |
| Strain | Supplement |  |  |  |
| EQ353 | No | 0.704286 | 0.015119 | [A1-1, A1-2, A1-3, C1, F1-1, F1-2, F1-3] |
| NCM3722 | 2 µg chloramphenicol | 0.790000 | NaN | [E1] |
|  | 4 µg chloramphenicol | 0.610000 | NaN | [E2] |
|  | 6 µg chloramphenicol | 0.490000 | NaN | [E3] |
|  | 8 µg chloramphenicol | 0.360000 | NaN | [E4] |
|  | No | 0.960000 | 0.033665 | [C2, A2, H1, H5] |
| NQ1243 | 100 µM 3MBA | 0.706667 | 0.015275 | [C4, C8, F5] |
|  | 400 µM 3MBA | 0.790000 | 0.028284 | [C3, F4] |
|  | No | 0.556667 | 0.005774 | [C5, D6, F6] |
| NQ1390 | 40 µM 3MBA | 0.340000 | 0.010000 | [C7, D7, F8] |
|  | 400 µM 3MBA | 0.490000 | 0.014142 | [C6, F7] |
|  | 400 µg 3MBA | 0.490000 | NaN | [D8] |
| NQ393 | 100 µM IPTG | 0.880000 | 0.036056 | [D5, F2, F3] |
|  | 20 µM IPTG | 0.220000 | NaN | [D1] |
|  | 30 µM IPTG | 0.360000 | NaN | [D2] |
|  | 40 µM IPTG | 0.540000 | NaN | [D3] |
|  | 50 µM IPTG | 0.670000 | NaN | [D4] |

The protein identifiers in the model start with "prot\_".

In [45]:

```
df_prot["model_id"] = df_prot["Protein ID"].apply(lambda prot: f"prot_{prot}")
```

Mark the proteins in the model to filter them during the condition selection.

This is not required (geckopy will ignore the unknown proteins) but will remove a lot of the warnings when applying protein concentrations of `from_mmol_gDW`.

In [46]:

```
df_prot["in_model"] = df_prot["model_id"].apply(lambda prot: prot in model.proteins)
```

In [47]:

```
df_prot.to_csv(DATA / "processed_proteins.tsv", sep="\t", index=False)
```

In [48]:

```
df_prot = pd.read_csv(DATA / "processed_proteins.tsv", sep="\t")
```

In addition to the number of proteins per sample per method, the primal (relaxation) values will be gathered in a dataframe for the LP first case to check how those are distributed and how numerical errors affect the solution.

In [49]:

```
df_results = pd.DataFrame({"sample": [], "protein": [], "primal": [], "dual": []})
df_results
```

Out[49]:

|  | sample | protein | primal | dual |
| --- | --- | --- | --- | --- |

For the conditions that exceed model unconstrained growth, the report growth will be lowered to the unconstrained maximum growth. Likely, the glucose uptake is greater than the one in the model but we do not have information about it.

In [50]:

```
unconstrained_growth = model.slim_optimize()
```

In [51]:

```
for i, (growth_mean, growth_std, sample) in tqdm(
    df_growth_agg_changes[["growth_mean", "growth_std", "sample"]].iterrows(),
    total=df_growth_agg_changes[["growth_mean", "growth_std", "sample"]].shape[0],
    desc="Agg samples IIS",
):
    if len(sample) > 1:
        df_sample = (
            df_prot.loc[
                df_prot.Sample.isin(sample) & df_prot.in_model, ["model_id", "mmolgDW"]
            ]
            .groupby("model_id")
            .agg([np.mean, np.std, "count"])
            .droplevel(0, axis=1)
            # use the upper limit of the CI at 0.95
            .assign(
                mmolgDW=lambda x: x["mean"] + 1.960 * x["std"] / np.sqrt(x["count"] - 1)
            )
            .reset_index()
        )
        # use the lower limit of the CI at 0.95
        growth = growth_mean - 1.960 * growth_std / sqrt(len(sample) - 1)
    else:
        df_sample = df_prot.loc[
            df_prot.Sample.isin(sample) & df_prot.in_model, ["model_id", "mmolgDW"]
        ]
        growth = growth_mean
    df_sample.index = df_sample["model_id"]
    # apply concentrations (this is not inplace)
    with warnings.catch_warnings():
        warnings.simplefilter("ignore")
        model_conc = from_mmol_gDW(model, df_sample["mmolgDW"])
    # force a growth condition before relaxing
    model_conc.reactions.get_by_id(objective_id).lower_bound = (
        growth if growth < unconstrained_growth else unconstrained_growth
    )
    prot_candidates = [
        prot.id for prot in model_conc.proteins if prot.concentration is not None
    ]
    prot_candidates = [
        prot_id
        for prot_id in prot_candidates
        if not isnan(model_conc.proteins.get_by_id(prot_id).concentration)
    ]
    iis_lp_agg = elastic_upper_relaxation(
        model_conc, prot_candidates, Objective_rule.MIN_ELASTIC_SUM
    )
    iis_milp_agg = elastic_upper_relaxation(
        model_conc, prot_candidates, Objective_rule.MIN_MILP_COUNT
    )
    iis_milp_agg_first, milp_status = get_upper_relaxation(
        model_conc.copy(), prot_candidates, Objective_rule.MIN_MILP_COUNT
    )
    iis_lp_agg_first, lp_status = get_upper_relaxation(
        model_conc, prot_candidates, Objective_rule.MIN_ELASTIC_SUM
    )
    iis_lp_dict_agg[str(sample)] = iis_lp_agg
    iis_milp_dict_agg[str(sample)] = iis_milp_agg
    iis_lp_dict_agg_first[str(sample)] = iis_lp_agg_first
    iis_milp_dict_agg_first[str(sample)] = iis_milp_agg_first
    # extract info for this sample
    if lp_status == "optimal":
        df_results = pd.concat(
            [
                df_results,
                pd.DataFrame(
                    {
                        "sample": len(iis_lp_agg_first) * [str(sample)],
                        "protein": [prot_id for prot_id in iis_lp_agg_first],
                        "primal": [
                            model_conc.variables.get(f"V_{prot_id}").primal
                            for prot_id in iis_lp_agg_first
                        ],
                        "dual": [
                            model_conc.variables.get(f"V_{prot_id}").dual
                            for prot_id in iis_lp_agg_first
                        ],
                        "method": "LP first",
                    }
                ),
            ]
        )
```

```
Agg samples IIS: 100%|██████████████████████████████████████████████████████████████████████████████████████████████████████████████████████████████████████| 17/17 [15:21<00:00, 54.21s/it]
```

In [52]:

```
tidy_results_agg = pd.DataFrame(
    {
        "protein": reduce(lambda a, b: list(a) + list(b), iis_lp_dict_agg.values(), []),
        "sample": reduce(
            lambda a, b: a + [b[0]] * len(b[1]), iis_lp_dict_agg.items(), []
        ),
    }
)
tidy_results_agg["method"] = "LP"
milp_results_agg = pd.DataFrame(
    {
        "protein": reduce(
            lambda a, b: list(a) + list(b), iis_milp_dict_agg.values(), []
        ),
        "sample": reduce(
            lambda a, b: a + [b[0]] * len(b[1]), iis_milp_dict_agg.items(), []
        ),
    }
)
milp_results_agg["method"] = "MILP"
tidy_results_agg_first = pd.DataFrame(
    {
        "protein": reduce(
            lambda a, b: list(a) + list(b), iis_lp_dict_agg_first.values(), []
        ),
        "sample": reduce(
            lambda a, b: a + [b[0]] * len(b[1]), iis_lp_dict_agg_first.items(), []
        ),
    }
)
tidy_results_agg_first["method"] = "LP first"
milp_results_agg_first = pd.DataFrame(
    {
        "protein": reduce(
            lambda a, b: list(a) + list(b), iis_milp_dict_agg_first.values(), []
        ),
        "sample": reduce(
            lambda a, b: a + [b[0]] * len(b[1]), iis_milp_dict_agg_first.items(), []
        ),
    }
)
milp_results_agg_first["method"] = "MILP first"
final_results_agg = pd.concat(
    [
        tidy_results_agg,
        milp_results_agg,
        tidy_results_agg_first,
        milp_results_agg_first,
    ],
    axis=0,
)
```

In [53]:

```
final_results_agg
```

Out[53]:

|  | protein | sample | method |
| --- | --- | --- | --- |
| 0 | prot\_P0AGG4 | ['A1-1', 'A1-2', 'A1-3', 'C1', 'F1-1', 'F1-2',... | LP |
| 1 | prot\_P69054 | ['A1-1', 'A1-2', 'A1-3', 'C1', 'F1-1', 'F1-2',... | LP |
| 2 | prot\_P08179 | ['A1-1', 'A1-2', 'A1-3', 'C1', 'F1-1', 'F1-2',... | LP |
| 3 | prot\_P21151 | ['A1-1', 'A1-2', 'A1-3', 'C1', 'F1-1', 'F1-2',... | LP |
| 4 | prot\_P08178 | ['A1-1', 'A1-2', 'A1-3', 'C1', 'F1-1', 'F1-2',... | LP |
| ... | ... | ... | ... |
| 1609 | prot\_P31119 | ['D4'] | MILP first |
| 1610 | prot\_P30138 | ['D4'] | MILP first |
| 1611 | prot\_P37306 | ['D4'] | MILP first |
| 1612 | prot\_P00562 | ['D4'] | MILP first |
| 1613 | prot\_P0AC16 | ['D4'] | MILP first |

5777 rows × 3 columns

In [54]:

```
final_results_agg.to_csv(
    DATA / "results_merged_allLP_AGG_wfirst.tsv", sep="\t", index=False
)
```

In [55]:

```
final_results_agg = pd.read_csv(DATA / "results_merged_allLP_AGG_wfirst.tsv", sep="\t")
```

In [56]:

```
df_results.head()
```

Out[56]:

|  | sample | protein | primal | dual | method |
| --- | --- | --- | --- | --- | --- |
| 0 | ['A1-1', 'A1-2', 'A1-3', 'C1', 'F1-1', 'F1-2',... | prot\_P21151 | 0.000049 | 0.0 | LP first |
| 1 | ['A1-1', 'A1-2', 'A1-3', 'C1', 'F1-1', 'F1-2',... | prot\_P0AEK2 | 0.000014 | 0.0 | LP first |
| 2 | ['A1-1', 'A1-2', 'A1-3', 'C1', 'F1-1', 'F1-2',... | prot\_P0A6I9 | 0.000002 | 0.0 | LP first |
| 3 | ['A1-1', 'A1-2', 'A1-3', 'C1', 'F1-1', 'F1-2',... | prot\_P0AFE4 | 0.000005 | 0.0 | LP first |
| 4 | ['A1-1', 'A1-2', 'A1-3', 'C1', 'F1-1', 'F1-2',... | prot\_P14407 | 0.000001 | 0.0 | LP first |

In [57]:

```
df_prot.head()
```

Out[57]:

|  | Protein ID | mmolgDW | MW | Sample | model\_id | in\_model |
| --- | --- | --- | --- | --- | --- | --- |
| 0 | P46482 | 0.000000e+00 | 34775.601615 | A1-1 | prot\_P46482 | False |
| 1 | P46481 | 0.000000e+00 | 73593.694590 | A1-1 | prot\_P46481 | False |
| 2 | P67662 | 0.000000e+00 | 34517.064180 | A1-1 | prot\_P67662 | False |
| 3 | P46478 | 0.000000e+00 | 7846.801510 | A1-1 | prot\_P46478 | False |
| 4 | P31119 | 2.713059e-07 | 80702.444030 | A1-1 | prot\_P31119 | True |

In [58]:

```
df_results_joined = pd.merge(df_results, df_prot[df_prot.in_model][["MW", "model_id"]].drop_duplicates(), how="left", left_on="protein", right_on="model_id")
```

In [59]:

```
df_results_joined["g/gdw"] = df_results_joined["primal"] * df_results_joined['MW'] / 1000
```

In [60]:

```
fig = px.bar(df_results_joined.groupby(["sample", "method"]).sum().reset_index(), x="sample", y ="g/gdw", width=800, height=600)
fig.update_layout(
    xaxis={"title": "Conditions"},
    yaxis={"title": "Total relaxation (g/gDW)"},
)

fig.update_xaxes(
    ticktext=ticktext,
    tickvals=tickvals,
)

fig.update_xaxes(titlefont=dict(size=21), tickfont=dict(size=16), tickangle=50)
fig.update_yaxes(titlefont=dict(size=21))
```

```
/tmp/ipykernel_60974/2918776753.py:1: FutureWarning: The default value of numeric_only in DataFrameGroupBy.sum is deprecated. In a future version, numeric_only will default to False. Either specify numeric_only or select only columns which should be valid for the function.
  fig = px.bar(df_results_joined.groupby(["sample", "method"]).sum().reset_index(), x="sample", y ="g/gdw", width=800, height=600)
```

In [61]:

```
df_prot["g/gdw"] = df_prot["mmolgDW"] * df_prot['MW'] / 1000
```

In [62]:

```
fig = px.bar(df_prot.groupby(["Sample"]).sum().reset_index(), x="Sample", y ="g/gdw", width=800, height=600)
fig.update_layout(
    xaxis={"title": "Conditions"},
    yaxis={"title": "Total Protein (g/gDW)"},
)


fig.update_xaxes(titlefont=dict(size=21), tickfont=dict(size=16), tickangle=50)
fig.update_yaxes(titlefont=dict(size=21))
```

```
/tmp/ipykernel_60974/2624582838.py:1: FutureWarning:

The default value of numeric_only in DataFrameGroupBy.sum is deprecated. In a future version, numeric_only will default to False. Either specify numeric_only or select only columns which should be valid for the function.
```

In [63]:

```
fig = px.bar(df_prot[df_prot.in_model].groupby(["Sample"]).sum().reset_index(), x="Sample", y ="g/gdw", width=800, height=600)
fig.update_layout(
    xaxis={"title": "Conditions"},
    yaxis={"title": "Total available protein (g/gDW)"},
)


fig.update_xaxes(titlefont=dict(size=21), tickfont=dict(size=16), tickangle=50)
fig.update_yaxes(titlefont=dict(size=21))
```

```
/tmp/ipykernel_60974/1951274844.py:1: FutureWarning:

The default value of numeric_only in DataFrameGroupBy.sum is deprecated. In a future version, numeric_only will default to False. Either specify numeric_only or select only columns which should be valid for the function.
```

In [64]:

```
df_results.to_csv("relaxation_output.tsv", sep="\t")
```

In [65]:

```
fig = px.histogram(
    final_results_agg.groupby(["protein", "method"])
    .count()
    .reset_index(drop=False)
    .rename({"sample": "appearances in samples"}, axis=1),
    x="appearances in samples",
    color="method",
    barmode="group",
    marginal="box",
    width=800,
    height=400,
    template="plotly_white",
)
fig.update_layout(
    legend=dict(
        x=0.1,
        y=0.5,
        traceorder="normal",
        font=dict(family="sans-serif", size=15, color="black"),
    )
)
fig.update_xaxes(titlefont=dict(size=21))
fig.update_yaxes(titlefont=dict(size=21))
fig
```

In [66]:

```
515 / 1259
```

Out[66]:

```
0.409054805401112
```

In [67]:

```
final_results_agg.groupby(["sample", "method"]).count().reset_index(drop=False).groupby(
    "method"
).agg([np.mean, np.std])
```

```
/tmp/ipykernel_60974/4187141397.py:1: FutureWarning:

['sample'] did not aggregate successfully. If any error is raised this will raise in a future version of pandas. Drop these columns/ops to avoid this warning.
```

Out[67]:

|  | protein | |
| --- | --- | --- |
|  | mean | std |
| method |  |  |
| LP | 75.176471 | 12.894937 |
| LP first | 75.823529 | 12.826902 |
| MILP | 93.882353 | 10.970884 |
| MILP first | 94.941176 | 11.415289 |

In [68]:

```
fig.write_image("results_0448_relax_proteo_mori2021_rep1.pdf")
```

In [69]:

```
set_prots = list(iis_milp_dict_agg_first.values())[0:2]
```

In [70]:

```
len(set_prots)
```

Out[70]:

```
2
```

#### Sensibility Aggregated by biological replicates¶

Generate the plot to inspect the relaxation on the LP first problem.

In [71]:

```
# save results
df_results.to_csv(
    DATA / "results_merged_LP_first_AGG_sens_with_cap.tsv", sep="\t", index=False
)
```

In [72]:

```
df_results = pd.read_csv(
    "relaxation_output.tsv", sep="\t"
)
```

In [73]:

```
def max_n(group):
    return group.loc[
        group["primal"] >= group["primal"].sort_values(ascending=False).iloc[10]
    ].sort_values("primal", ascending=False)


def more_than(group, than=1e-7):
    return group.loc[group["primal"] >= than].sort_values("primal", ascending=False)
```

In [74]:

```
df_results_top10_sample = (
    df_results.groupby("sample").apply(max_n).reset_index(drop=True)
)
```

In [75]:

```
fig_lp_sample_more_than = px.bar(
    df_results_top10_sample,
    color="protein",
    y="primal",
    x="sample",
    template="plotly_white",
)

fig_lp_sample_more_than
```

In [76]:

```
df_results_top10_sample = (
    df_results.groupby("sample").apply(max_n).reset_index(drop=True)
)
df_results_top10_sample["sample"] = df_results_top10_sample["sample"].replace(
    {s: i for i, s in enumerate(df_results_top10_sample["sample"].unique())}
)
```

In [77]:

```
df_results_top10_sample
```

Out[77]:

|  | Unnamed: 0 | sample | protein | primal | dual | method |
| --- | --- | --- | --- | --- | --- | --- |
| 0 | 39 | 0 | prot\_P15254 | 0.001688 | 0.0 | LP first |
| 1 | 64 | 0 | prot\_P0AB98 | 0.000690 | 0.0 | LP first |
| 2 | 57 | 0 | prot\_P0A6E6 | 0.000686 | 0.0 | LP first |
| 3 | 78 | 0 | prot\_P0ABA0 | 0.000681 | 0.0 | LP first |
| 4 | 60 | 0 | prot\_P0ABA4 | 0.000676 | 0.0 | LP first |
| ... | ... | ... | ... | ... | ... | ... |
| 182 | 10 | 16 | prot\_P76503 | 0.000116 | 0.0 | LP first |
| 183 | 2 | 16 | prot\_P68699 | 0.000104 | 0.0 | LP first |
| 184 | 28 | 16 | prot\_P06959 | 0.000098 | 0.0 | LP first |
| 185 | 5 | 16 | prot\_P0AFG8 | 0.000092 | 0.0 | LP first |
| 186 | 53 | 16 | prot\_P32669 | 0.000091 | 0.0 | LP first |

187 rows × 6 columns

In [78]:

```
fig_lp_sample_more_than = px.bar(
    df_results_top10_sample,
    color="protein",
    y="primal",
    x="sample",
    template="plotly_white",
)

fig_lp_sample_more_than
```

In [79]:

```
fig_lp_sample_more_than.write_image(
    "results_0448_relax_proteo_mori2021_sample_more_than_lpfirst.pdf"
)
```

In [80]:

```
model.reactions.get_by_id("PRFGSNo1")
```

Out[80]:

|  |  |
| --- | --- |
| **Reaction identifier** | PRFGSNo1 |
| **Name** | Phosphoribosylformylglycinamidine synthase (No1) |
| **Memory address** | 0x07ff20f3d6e80 |
| **Stoichiometry** | atp\_c + fgam\_c + gln\_\_L\_c + h2o\_c + 0.0055556 prot\_P15254 --> adp\_c + fpram\_c + glu\_\_L\_c + h\_c + pi\_c  ATP [cytosol] + N2-Formyl-N1-(5-phospho-D-ribosyl)glycinamide [cytosol] + L-Glutamine [cytosol] + H2O [cytosol] + 0.0055556 prot\_P15254 [cytosol] --> ADP [cytosol] + 2-(Formamido)-N1-(5-phospho-D-... |
| **GPR** | b2557 |
| **Lower bound** | 0.0 |
| **Upper bound** | 1000.0 |

In [81]:

```
model.proteins.prot_P0ABA6.kcats
```

Out[81]:

```
{<Reaction ATPS4rppNo2 at 0x7ff20fe74b80>: 19.999840001279992, <Reaction ATPS4rpp_REVNo1 at 0x7ff20e983d90>: 284.99966940038354, <Reaction ATPS4rpp_REVNo2 at 0x7ff20e993b20>: 284.99966940038354, <Reaction ATPS4rppNo1 at 0x7ff20fe04eb0>: 19.999840001279992}
```

In [82]:

```
{
    prot: [reac.name for reac in model.proteins.get_by_id(prot).reactions]
    for prot in df_results_top10_sample.loc[
        df_results_top10_sample["sample"] == 20, "protein"
    ].to_list()
}
```

Out[82]:

```
{}
```

In [83]:

```
df_results_more_than_sample = (
    df_results.groupby("sample")
    .apply(lambda x: more_than(x, 1e-7))
    .reset_index(drop=True)
)
```

In [84]:

```
df_results_more_than_sample.groupby("sample").count().mean()
```

Out[84]:

```
Unnamed: 0    74.058824
protein       74.058824
primal        74.058824
dual          74.058824
method        74.058824
dtype: float64
```

In [85]:

```
df_growth_agg_changes
```

Out[85]:

|  |  | growth\_mean | growth\_std | sample |
| --- | --- | --- | --- | --- |
| Strain | Supplement |  |  |  |
| EQ353 | No | 0.704286 | 0.015119 | [A1-1, A1-2, A1-3, C1, F1-1, F1-2, F1-3] |
| NCM3722 | 2 µg chloramphenicol | 0.790000 | NaN | [E1] |
|  | 4 µg chloramphenicol | 0.610000 | NaN | [E2] |
|  | 6 µg chloramphenicol | 0.490000 | NaN | [E3] |
|  | 8 µg chloramphenicol | 0.360000 | NaN | [E4] |
|  | No | 0.960000 | 0.033665 | [C2, A2, H1, H5] |
| NQ1243 | 100 µM 3MBA | 0.706667 | 0.015275 | [C4, C8, F5] |
|  | 400 µM 3MBA | 0.790000 | 0.028284 | [C3, F4] |
|  | No | 0.556667 | 0.005774 | [C5, D6, F6] |
| NQ1390 | 40 µM 3MBA | 0.340000 | 0.010000 | [C7, D7, F8] |
|  | 400 µM 3MBA | 0.490000 | 0.014142 | [C6, F7] |
|  | 400 µg 3MBA | 0.490000 | NaN | [D8] |
| NQ393 | 100 µM IPTG | 0.880000 | 0.036056 | [D5, F2, F3] |
|  | 20 µM IPTG | 0.220000 | NaN | [D1] |
|  | 30 µM IPTG | 0.360000 | NaN | [D2] |
|  | 40 µM IPTG | 0.540000 | NaN | [D3] |
|  | 50 µM IPTG | 0.670000 | NaN | [D4] |

In [86]:

```
print(
    f"Relaxation results: {len(df_results_more_than_sample['sample'].unique())}\n"
    f"Ticktext len: {len(ticktext)}\n"
    f"Tickvals len: {len(tickvals)}\n"
)
```

```
Relaxation results: 17
Ticktext len: 17
Tickvals len: 17
```

In [87]:

```
fig = px.bar(
    df_results_more_than_sample,
    color="protein",
    y="primal",
    x="sample",
    hover_data=["sample"],
    width=1200,
    height=800,
    template="plotly_white",
)

fig.update_layout(
    xaxis={"title": "Conditions"},
    yaxis={"title": "relaxation primal value (> 1e-7)"},
)

fig.update_xaxes(
    ticktext=ticktext,
    tickvals=tickvals,
)

fig.update_xaxes(titlefont=dict(size=21), tickfont=dict(size=16), tickangle=50)
fig.update_yaxes(titlefont=dict(size=21))
```

In [88]:

```
model.proteins.prot_P0AGB0.kcats["PSP_LNo1"]
```

Out[88]:

```
0.14999610010139736
```

In [89]:

```
model.reactions.PSP_LNo1
```

Out[89]:

|  |  |
| --- | --- |
| **Reaction identifier** | PSP\_LNo1 |
| **Name** | Phosphoserine phosphatase (L-serine) (No1) |
| **Memory address** | 0x07ff20f349c40 |
| **Stoichiometry** | h2o\_c + 0.0018519 prot\_P0AGB0 + pser\_\_L\_c --> pi\_c + ser\_\_L\_c  H2O [cytosol] + 0.0018519 prot\_P0AGB0 [cytosol] + O-Phospho-L-serine [cytosol] --> Phosphate [cytosol] + L-Serine [cytosol] |
| **GPR** | b4388 |
| **Lower bound** | 0.0 |
| **Upper bound** | 1000.0 |

In [90]:

```
sum(model.proteins.prot_P0AEN1.kcats.values()) / len(
    model.proteins.prot_P0AEN1.kcats.items()
)
```

Out[90]:

```
0.16800397833420697
```

In [91]:

```
model.reactions.FMNRxNo2
```

Out[91]:

|  |  |
| --- | --- |
| **Reaction identifier** | FMNRxNo2 |
| **Name** | FMN reductase (No2) |
| **Memory address** | 0x07ff20eec2ee0 |
| **Stoichiometry** | pmet\_FMNRx + 0.0016534 prot\_P0AEN1 --> fmnh2\_c + nad\_c  pmet\_FMNRx [cytosol] + 0.0016534 prot\_P0AEN1 [cytosol] --> Reduced FMN [cytosol] + NAD+ [cytosol] |
| **GPR** | b3844 |
| **Lower bound** | 0.0 |
| **Upper bound** | 1000.0 |

In [92]:

```
fig.write_image("results_0448_relax_proteo_mori2021_sample_more_than_lpfirst.pdf")
```

Linear fit of total relaxation ~ growth rate.

In [93]:

```
df_growth_agg_changes
```

Out[93]:

|  |  | growth\_mean | growth\_std | sample |
| --- | --- | --- | --- | --- |
| Strain | Supplement |  |  |  |
| EQ353 | No | 0.704286 | 0.015119 | [A1-1, A1-2, A1-3, C1, F1-1, F1-2, F1-3] |
| NCM3722 | 2 µg chloramphenicol | 0.790000 | NaN | [E1] |
|  | 4 µg chloramphenicol | 0.610000 | NaN | [E2] |
|  | 6 µg chloramphenicol | 0.490000 | NaN | [E3] |
|  | 8 µg chloramphenicol | 0.360000 | NaN | [E4] |
|  | No | 0.960000 | 0.033665 | [C2, A2, H1, H5] |
| NQ1243 | 100 µM 3MBA | 0.706667 | 0.015275 | [C4, C8, F5] |
|  | 400 µM 3MBA | 0.790000 | 0.028284 | [C3, F4] |
|  | No | 0.556667 | 0.005774 | [C5, D6, F6] |
| NQ1390 | 40 µM 3MBA | 0.340000 | 0.010000 | [C7, D7, F8] |
|  | 400 µM 3MBA | 0.490000 | 0.014142 | [C6, F7] |
|  | 400 µg 3MBA | 0.490000 | NaN | [D8] |
| NQ393 | 100 µM IPTG | 0.880000 | 0.036056 | [D5, F2, F3] |
|  | 20 µM IPTG | 0.220000 | NaN | [D1] |
|  | 30 µM IPTG | 0.360000 | NaN | [D2] |
|  | 40 µM IPTG | 0.540000 | NaN | [D3] |
|  | 50 µM IPTG | 0.670000 | NaN | [D4] |

In [94]:

```
len(df_results_more_than_sample["sample"].unique())
```

Out[94]:

```
17
```

In [95]:

```
len(df_growth_agg_changes.assign(
        sample=df_growth_agg_changes["sample"].astype(str)
    )["sample"].unique())
```

Out[95]:

```
17
```

Gather the Mw to express the sum of relaxation is mass units.

In [96]:

```
df_prot = pd.read_csv(DATA / "processed_proteins.tsv", sep="\t")
```

In [97]:

```
df_results_more_than_sample = pd.merge(df_results_more_than_sample, df_prot[df_prot.in_model][["MW", "model_id"]].drop_duplicates(), how="left", left_on="protein", right_on="model_id")
```

In [98]:

```
df_results_more_than_sample["g/gDW"] = df_results_more_than_sample.MW * df_results_more_than_sample.primal
```

In [99]:

```
df_results_more_than_sample
```

Out[99]:

|  | Unnamed: 0 | sample | protein | primal | dual | method | MW | model\_id | g/gDW |
| --- | --- | --- | --- | --- | --- | --- | --- | --- | --- |
| 0 | 39 | ['A1-1', 'A1-2', 'A1-3', 'C1', 'F1-1', 'F1-2',... | prot\_P15254 | 1.688439e-03 | 0.0 | LP first | 21039.510560 | prot\_P15254 | 35.523920 |
| 1 | 64 | ['A1-1', 'A1-2', 'A1-3', 'C1', 'F1-1', 'F1-2',... | prot\_P0AB98 | 6.898780e-04 | 0.0 | LP first | 15068.747730 | prot\_P0AB98 | 10.395597 |
| 2 | 57 | ['A1-1', 'A1-2', 'A1-3', 'C1', 'F1-1', 'F1-2',... | prot\_P0A6E6 | 6.858945e-04 | 0.0 | LP first | 50326.998965 | prot\_P0A6E6 | 34.519014 |
| 3 | 78 | ['A1-1', 'A1-2', 'A1-3', 'C1', 'F1-1', 'F1-2',... | prot\_P0ABA0 | 6.808795e-04 | 0.0 | LP first | 31578.427810 | prot\_P0ABA0 | 21.501104 |
| 4 | 60 | ['A1-1', 'A1-2', 'A1-3', 'C1', 'F1-1', 'F1-2',... | prot\_P0ABA4 | 6.760026e-04 | 0.0 | LP first | 13632.140275 | prot\_P0ABA4 | 9.215362 |
| ... | ... | ... | ... | ... | ... | ... | ... | ... | ... |
| 1254 | 58 | ['E4'] | prot\_P37306 | 1.964230e-07 | 0.0 | LP first | 28790.670620 | prot\_P37306 | 0.005655 |
| 1255 | 61 | ['E4'] | prot\_P0AC16 | 1.790361e-07 | 0.0 | LP first | 45407.127735 | prot\_P0AC16 | 0.008130 |
| 1256 | 3 | ['E4'] | prot\_P08178 | 1.483041e-07 | 0.0 | LP first | 33994.393370 | prot\_P08178 | 0.005042 |
| 1257 | 54 | ['E4'] | prot\_P0A9U8 | 1.423506e-07 | 0.0 | LP first | 26726.217655 | prot\_P0A9U8 | 0.003804 |
| 1258 | 4 | ['E4'] | prot\_P0A853 | 1.291880e-07 | 0.0 | LP first | 9094.835400 | prot\_P0A853 | 0.001175 |

1259 rows × 9 columns

In [100]:

```
growth_total_primal = pd.merge(
    df_results_more_than_sample.groupby("sample")["g/gDW"].sum().reset_index(),
    df_growth_agg_changes[["sample", "growth_mean"]].assign(
        sample=df_growth_agg_changes["sample"].astype(str)
    ),
    on="sample",
)
```

In [101]:

```
x = growth_total_primal["g/gDW"].to_numpy()
y = growth_total_primal.growth_mean.to_numpy()
```

In [102]:

```
tick_to_condition = dict(zip(tickvals, ticktext))
```

In [103]:

```
conditions = [tick_to_condition[x] for x in growth_total_primal["sample"]]
```

In [104]:

```
conditions = [cond.split(" ")[0] for cond in conditions]
```

In [105]:

```
color_map = {"Reference": "*", "R-limitation": "o", "C-limitation": "D", "A-limitation": "^"}
```

In [106]:

```
from scipy.stats import pearsonr
```

In [107]:

```
pearsonr(x, y)
```

Out[107]:

```
PearsonRResult(statistic=0.950865751921021, pvalue=4.874810172602218e-09)
```

In [108]:

```
m, c = np.linalg.lstsq(np.vstack([x, np.ones(len(x))]).T, y, rcond=None)[0]
```

In [109]:

```
import matplotlib.pyplot as plt

plt.rcParams["figure.figsize"] = (16, 10)
plt.rcParams.update({"font.size": 22})
```

In [110]:

```
plt.style.use("seaborn-whitegrid")
```

```
/tmp/ipykernel_60974/3963306264.py:1: MatplotlibDeprecationWarning:

The seaborn styles shipped by Matplotlib are deprecated since 3.6, as they no longer correspond to the styles shipped by seaborn. However, they will remain available as 'seaborn-v0_8-<style>'. Alternatively, directly use the seaborn API instead.
```

In [111]:

```
unconstrained_growth = model.slim_optimize()
```

In [112]:

```
_ = plt.plot(
    x[y > unconstrained_growth],
    y[y > unconstrained_growth],
    "s",
    color="#00cc96",
    label="Exceeding model growth",
    markersize=15,
)
#_ = plt.plot(x, y, "o", color="#636efa", label="Original data", markersize=10)
for name, group in pd.DataFrame({"x": x, "y": y, "conditions": conditions}).groupby("conditions"):
    if name == "Reference":
        _ = plt.plot(group.x, group.y, color_map[name], color="black", label=name, markersize=10)
    else:
        _ = plt.plot(group.x, group.y, color_map[name], label=name, markersize=10)

_ = plt.plot(x, m * x + c, color="#ef553b", label="Fitted line")
plt.xlabel("Sum of relaxation ($g/gDW$)")
plt.ylabel("Growth rate ($h^{-1}$)")
_ = plt.legend(frameon=True, edgecolor="white")
#plt.tight_layout()
plt.savefig("fitting_growth_relaxation.pdf", dpi=300, bbox_inches="tight")
plt.savefig("fitting_growth_relaxation.eps", dpi=300, bbox_inches="tight")
plt.show()
```

```
The PostScript backend does not support transparency; partially transparent artists will be rendered opaque.
```

In [113]:

```
df_results_more_than_sample.groupby("sample").count()["protein"].agg(["mean", "std"])
```

Out[113]:

```
mean    74.058824
std     13.418600
Name: protein, dtype: float64
```

In [114]:

```
df_results_more_than6_sample = (
    df_results.groupby("sample")
    .apply(lambda x: more_than(x, 1e-6))
    .reset_index(drop=True)
)
```

In [115]:

```
fig = px.histogram(
    df_results_more_than6_sample.groupby(["protein"]).count().reset_index(drop=False),
    x="sample",
    barmode="group",
    marginal="box",
    width=800,
    height=400,
    template="plotly_white",
)
fig.update_layout(
    legend=dict(
        x=0.1,
        y=0.5,
        traceorder="normal",
        font=dict(family="sans-serif", size=12, color="black"),
    )
)
fig
```

## Get average enzyme saturation¶

In [116]:

```
from geckopy.flux_analysis import (
    count_protein_saturation,
    get_average_protein_saturation,
)
```

In [117]:

```
growth_conditions = pd.read_excel(
    DATA / "msb20209536-sup-0004-datasetev3.xlsx", sheet_name=1, engine="openpyxl"
)
```

In [118]:

```
growth_conditions.Supplement = growth_conditions.Supplement.apply(lambda x: "No" if pd.isna(x) else x)
# group growth conditions by biological replicates
df_growth_agg_changes = growth_conditions.groupby(["Strain", "Supplement"]).agg(
    growth_mean=pd.NamedAgg(column="Growth rate (1/h)", aggfunc="mean"),
    growth_std=pd.NamedAgg(column="Growth rate (1/h)", aggfunc="std"),
    sample=pd.NamedAgg(column="Sample ID", aggfunc=lambda x: x.to_list()),
)
# add info to later delete gldha on NQ393
df_growth_agg_changes["gdha_del"] = (
    df_growth_agg_changes.reset_index().Strain.str.match("NQ393").to_list()
)
```

In [119]:

```
df_prot = pd.read_csv(DATA / "processed_proteins.tsv", sep="\t")
```

In [120]:

```
model = geckopy.io.read_sbml_ec_model(DATA / "eciML1515.xml.gz")
```

In [121]:

```
model.solver = "cplex"
objective_id = "BIOMASS_Ec_iML1515_core_75p37M"
unconstrained_growth = model.slim_optimize()
```

In [122]:

```
df_results = pd.DataFrame({"sample": [], "count": [], "average": [], "method": []})
df_results
```

Out[122]:

|  | sample | count | average | method |
| --- | --- | --- | --- | --- |

In [123]:

```
df_results_long = pd.DataFrame({"sample": [], "method": [], "contribution": [], "upper_bound": [], "protein": []})
```

In [124]:

```
df_prot.head()
```

Out[124]:

|  | Protein ID | mmolgDW | MW | Sample | model\_id | in\_model |
| --- | --- | --- | --- | --- | --- | --- |
| 0 | P46482 | 0.000000e+00 | 34775.601615 | A1-1 | prot\_P46482 | False |
| 1 | P46481 | 0.000000e+00 | 73593.694590 | A1-1 | prot\_P46481 | False |
| 2 | P67662 | 0.000000e+00 | 34517.064180 | A1-1 | prot\_P67662 | False |
| 3 | P46478 | 0.000000e+00 | 7846.801510 | A1-1 | prot\_P46478 | False |
| 4 | P31119 | 2.713059e-07 | 80702.444030 | A1-1 | prot\_P31119 | True |

We want the average protein usage but also the average protein usage of active (non-zero drain) enzymes. Thus, we can define a function to get the full distribution and then plot it as we prefer.

In [125]:

```
def get_protein_saturation(model: geckopy.Model) -> list[tuple[str, float, float]]:
    """Compute the average upper bounds usage by the proteins in the model."""
    non_zero_prots = [prot for prot in model.proteins if prot.upper_bound]
    return [(prot.id, prot.contribution, prot.concentration) for prot in non_zero_prots]
```

In [126]:

```
for i, (growth_mean, growth_std, sample, gdha_del) in tqdm(
    df_growth_agg_changes[
        ["growth_mean", "growth_std", "sample", "gdha_del"]
    ].iterrows(),
    total=df_growth_agg_changes[["growth_mean", "growth_std", "sample"]].shape[0],
    desc="Agg samples sensibility IIS",
):
    if len(sample) > 1:
        # group the dataframe by biological replicates (given by growth conditions)
        df_sample = (
            df_prot.loc[
                df_prot.Sample.isin(sample) & df_prot.in_model, ["model_id", "mmolgDW"]
            ]
            .groupby("model_id")
            .agg([np.mean, np.std, "count"])
            .droplevel(0, axis=1)
            # use the upper limit of the CI at 0.95
            .assign(
                mmolgDW=lambda x: x["mean"] + 1.960 * x["std"] / np.sqrt(x["count"] - 1)
            )
            .reset_index()
        )
        # use the lower limit of the CI at 0.95
        growth = growth_mean - 1.960 * growth_std / sqrt(len(sample) - 1)
    else:
        df_sample = df_prot.loc[
            df_prot.Sample.isin(sample) & df_prot.in_model, ["model_id", "mmolgDW"]
        ]
        growth = growth_mean
    df_sample.index = df_sample["model_id"]
    # apply concentrations (this is not inplace)
    with warnings.catch_warnings():
        warnings.simplefilter("ignore")
        model_conc = from_mmol_gDW(model, df_sample["mmolgDW"])
    # remove gdha
    if gdha_del:
        model_conc.reactions.GLUDy_REVNo1.bounds = 0, 0
        model_conc.reactions.GLUDyNo1.bounds = 0, 0
    # force a growth condition before relaxing
    model_conc.reactions.get_by_id(objective_id).lower_bound = (
        growth if growth < unconstrained_growth else unconstrained_growth
    )
    prot_candidates = [
        prot.id for prot in model_conc.proteins if prot.concentration is not None
    ]
    prot_candidates = [
        prot_id
        for prot_id in prot_candidates
        if not isnan(model_conc.proteins.get_by_id(prot_id).concentration)
    ]
    model_elastic = model_conc.copy()
    _, lp_status = get_upper_relaxation(
        model_elastic, prot_candidates, Objective_rule.MIN_ELASTIC_SUM
    )
    _ = get_upper_relaxation(model_conc, prot_candidates, Objective_rule.MIN_MILP_COUNT)
    # extract info for this sample
    if lp_status == "optimal":
        df_results = pd.concat(
            [
                df_results,
                pd.DataFrame(
                    {
                        "sample": 2 * [str(sample)],
                        "count": [
                            count_protein_saturation(model_elastic),
                            count_protein_saturation(model_conc),
                        ],
                        "average": [
                            get_average_protein_saturation(model_elastic),
                            get_average_protein_saturation(model_conc),
                        ],
                        "method": [
                            "LP first",
                            "MILP first",
                        ],
                    }
                ),
            ]
        )
        saturation_lp = get_protein_saturation(model_elastic)
        saturation_milp = get_protein_saturation(model_conc)
        df_results_long = pd.concat(
            [
                df_results_long,
                pd.DataFrame(
                    {
                        "sample": str(sample),
                        "contribution": [val[1] for val in saturation_lp + saturation_milp],
                        "upper_bound": [val[2] for val in saturation_lp + saturation_milp],
                        "protein": [val[0] for val in saturation_lp + saturation_milp],
                        "method": ["LP first"] * len(saturation_lp)
                        + ["MILP first"] * len(saturation_milp),
                    }
                ),
            ]
        )
```

```
Agg samples sensibility IIS: 100%|██████████████████████████████████████████████████████████████████████████████████████████████████████████████████████████| 17/17 [05:14<00:00, 18.47s/it]
```

In [127]:

```
df_results_long["saturation"] = df_results_long.contribution / df_results_long.upper_bound
```

In [128]:

```
df_results_long.to_csv("contribution_and_saturation_first_methods.tsv", sep="\t", index=False)
```

In [129]:

```
df_results_long = pd.read_csv("contribution_and_saturation_first_methods.tsv", sep="\t")
```

In [130]:

```
df_results_long.head()
```

Out[130]:

|  | sample | method | contribution | upper\_bound | protein | saturation |
| --- | --- | --- | --- | --- | --- | --- |
| 0 | ['A1-1', 'A1-2', 'A1-3', 'C1', 'F1-1', 'F1-2',... | LP first | 2.363062e-05 | 9.243180e-05 | prot\_P0A825 | 0.255655 |
| 1 | ['A1-1', 'A1-2', 'A1-3', 'C1', 'F1-1', 'F1-2',... | LP first | 0.000000e+00 | 1.377791e-06 | prot\_P75823 | 0.000000 |
| 2 | ['A1-1', 'A1-2', 'A1-3', 'C1', 'F1-1', 'F1-2',... | LP first | 3.385025e-09 | 4.845740e-07 | prot\_P0AEA8 | 0.006986 |
| 3 | ['A1-1', 'A1-2', 'A1-3', 'C1', 'F1-1', 'F1-2',... | LP first | 1.183178e-07 | 3.434312e-06 | prot\_P06715 | 0.034452 |
| 4 | ['A1-1', 'A1-2', 'A1-3', 'C1', 'F1-1', 'F1-2',... | LP first | 7.395810e-07 | 1.619744e-05 | prot\_P0A7E1 | 0.045660 |

In [131]:

```
df_results_long
```

Out[131]:

|  | sample | method | contribution | upper\_bound | protein | saturation |
| --- | --- | --- | --- | --- | --- | --- |
| 0 | ['A1-1', 'A1-2', 'A1-3', 'C1', 'F1-1', 'F1-2',... | LP first | 2.363062e-05 | 9.243180e-05 | prot\_P0A825 | 0.255655 |
| 1 | ['A1-1', 'A1-2', 'A1-3', 'C1', 'F1-1', 'F1-2',... | LP first | 0.000000e+00 | 1.377791e-06 | prot\_P75823 | 0.000000 |
| 2 | ['A1-1', 'A1-2', 'A1-3', 'C1', 'F1-1', 'F1-2',... | LP first | 3.385025e-09 | 4.845740e-07 | prot\_P0AEA8 | 0.006986 |
| 3 | ['A1-1', 'A1-2', 'A1-3', 'C1', 'F1-1', 'F1-2',... | LP first | 1.183178e-07 | 3.434312e-06 | prot\_P06715 | 0.034452 |
| 4 | ['A1-1', 'A1-2', 'A1-3', 'C1', 'F1-1', 'F1-2',... | LP first | 7.395810e-07 | 1.619744e-05 | prot\_P0A7E1 | 0.045660 |
| ... | ... | ... | ... | ... | ... | ... |
| 27649 | ['D4'] | MILP first | 0.000000e+00 | 2.247169e-07 | prot\_P16689 | 0.000000 |
| 27650 | ['D4'] | MILP first | 0.000000e+00 | NaN | prot\_P0DP69 | NaN |
| 27651 | ['D4'] | MILP first | 0.000000e+00 | 1.397438e-06 | prot\_P75728 | 0.000000 |
| 27652 | ['D4'] | MILP first | 0.000000e+00 | 8.212228e-07 | prot\_P25535 | 0.000000 |
| 27653 | ['D4'] | MILP first | 0.000000e+00 | 1.010036e-06 | prot\_P76290 | 0.000000 |

27654 rows × 6 columns

In [132]:

```
df_results_long["reaction"] = df_results_long.protein.apply(lambda x: [reac.id for reac in model.proteins.get_by_id(x).reactions])
```

In [133]:

```
df_results_reactions = df_results_long.explode("reaction")
```

In [134]:

```
df_results_reactions.to_csv("contribution_and_saturation_first_methods_reactions.tsv", sep="\t", index=False)
```

In [135]:

```
df_results_reactions = pd.read_csv("contribution_and_saturation_first_methods_reactions.tsv", sep="\t")
```

In [136]:

```
df_results_reactions
```

Out[136]:

|  | sample | method | contribution | upper\_bound | protein | saturation | reaction |
| --- | --- | --- | --- | --- | --- | --- | --- |
| 0 | ['A1-1', 'A1-2', 'A1-3', 'C1', 'F1-1', 'F1-2',... | LP first | 0.000024 | 9.243180e-05 | prot\_P0A825 | 0.255655 | THFATNo1 |
| 1 | ['A1-1', 'A1-2', 'A1-3', 'C1', 'F1-1', 'F1-2',... | LP first | 0.000024 | 9.243180e-05 | prot\_P0A825 | 0.255655 | ALATA\_L2No2 |
| 2 | ['A1-1', 'A1-2', 'A1-3', 'C1', 'F1-1', 'F1-2',... | LP first | 0.000024 | 9.243180e-05 | prot\_P0A825 | 0.255655 | GHMT2rNo1 |
| 3 | ['A1-1', 'A1-2', 'A1-3', 'C1', 'F1-1', 'F1-2',... | LP first | 0.000024 | 9.243180e-05 | prot\_P0A825 | 0.255655 | GHMT2r\_REVNo1 |
| 4 | ['A1-1', 'A1-2', 'A1-3', 'C1', 'F1-1', 'F1-2',... | LP first | 0.000024 | 9.243180e-05 | prot\_P0A825 | 0.255655 | THRANo1 |
| ... | ... | ... | ... | ... | ... | ... | ... |
| 81689 | ['D4'] | MILP first | 0.000000 | 2.247169e-07 | prot\_P16689 | 0.000000 | RPNTPHNo1 |
| 81690 | ['D4'] | MILP first | 0.000000 | NaN | prot\_P0DP69 | NaN | MEPNabcppNo1 |
| 81691 | ['D4'] | MILP first | 0.000000 | 1.397438e-06 | prot\_P75728 | 0.000000 | OMMBLHXyNo1 |
| 81692 | ['D4'] | MILP first | 0.000000 | 8.212228e-07 | prot\_P25535 | 0.000000 | OPHHXyNo1 |
| 81693 | ['D4'] | MILP first | 0.000000 | 1.010036e-06 | prot\_P76290 | 0.000000 | CXSAMSNo1 |

81694 rows × 7 columns

In [137]:

```
sample_to_condition = dict(zip(tickvals, ticktext))
```

In [ ]:

```

```

In [138]:

```
df_results_reactions["condition"] = df_results_reactions["sample"].apply(lambda x: sample_to_condition[x])
```

In [139]:

```
df_results_reactions = df_results_reactions.groupby(["condition", "method", "reaction"]).sum().reset_index()
```

```
/tmp/ipykernel_60974/2758412221.py:1: FutureWarning:

The default value of numeric_only in DataFrameGroupBy.sum is deprecated. In a future version, numeric_only will default to False. Either specify numeric_only or select only columns which should be valid for the function.
```

In [140]:

```
df_results_reactions.head()
```

Out[140]:

|  | condition | method | reaction | contribution | upper\_bound | saturation |
| --- | --- | --- | --- | --- | --- | --- |
| 0 | A-limitation (100 µM IPTG) NQ393 | LP first | 13PPDH2No1 | 0.0 | 0.000000e+00 | 0.0 |
| 1 | A-limitation (100 µM IPTG) NQ393 | LP first | 13PPDH2\_REVNo1 | 0.0 | 0.000000e+00 | 0.0 |
| 2 | A-limitation (100 µM IPTG) NQ393 | LP first | 14GLUCANabcppNo1 | 0.0 | 6.762505e-07 | 0.0 |
| 3 | A-limitation (100 µM IPTG) NQ393 | LP first | 23PDE2ppNo1 | 0.0 | 6.883129e-07 | 0.0 |
| 4 | A-limitation (100 µM IPTG) NQ393 | LP first | 23PDE4ppNo1 | 0.0 | 6.883129e-07 | 0.0 |

In [141]:

```
df_results_reactions.loc[df_results_reactions.saturation > 1, "saturation"] = 1.0
df_results_reactions.loc[df_results_reactions.saturation <= 0, "saturation"] = 0.0
```

In [142]:

```
df_results_long.saturation = df_results_long.contribution / df_results_long.upper_bound
```

There may be enzymes with saturation > 1 because of numerical errors.

In [143]:

```
excess_results = df_results_long[df_results_long.upper_bound < df_results_long.contribution]
```

In [144]:

```
excess_results.shape
```

Out[144]:

```
(76, 7)
```

Are these 83 enzymes below the numerical error threshold ~10e-7?

In [145]:

```
abs(excess_results.upper_bound - excess_results.contribution).describe()
```

Out[145]:

```
count    7.600000e+01
mean     1.754181e-07
std      2.198697e-07
min      1.690805e-09
25%      4.107102e-08
50%      7.134707e-08
75%      2.574790e-07
max      9.978674e-07
dtype: float64
```

In [146]:

```
excess_results.contribution.describe()
```

Out[146]:

```
count    7.600000e+01
mean     1.449379e-06
std      1.745768e-06
min      1.937423e-08
25%      4.538717e-07
50%      7.587360e-07
75%      1.280331e-06
max      7.036671e-06
Name: contribution, dtype: float64
```

Let's cap the saturation and then check the contribution and saturation distributions.

In [147]:

```
df_results_long.loc[df_results_long.saturation > 1, "saturation"] = 1.0
```

In [148]:

```
df_results_long = df_results_long[df_results_long.contribution > 1e-7]
```

In [149]:

```
df_results_long.describe()
```

Out[149]:

|  | contribution | upper\_bound | saturation |
| --- | --- | --- | --- |
| count | 8.078000e+03 | 8.061000e+03 | 8061.000000 |
| mean | 6.747821e-06 | 1.155321e-04 | 0.504163 |
| std | 1.772671e-05 | 1.491889e-03 | 0.413778 |
| min | 1.001784e-07 | 7.844415e-08 | 0.000880 |
| 25% | 4.545389e-07 | 1.913798e-06 | 0.089569 |
| 50% | 1.494415e-06 | 5.895311e-06 | 0.386250 |
| 75% | 5.055871e-06 | 1.827789e-05 | 1.000000 |
| max | 2.492020e-04 | 3.582262e-02 | 1.000000 |

In [150]:

```
df_results_over_0 = df_results_long.groupby(["sample", "method"]).saturation.mean().reset_index()
```

In [151]:

```
df_results_over_0.head()
```

Out[151]:

|  | sample | method | saturation |
| --- | --- | --- | --- |
| 0 | ['A1-1', 'A1-2', 'A1-3', 'C1', 'F1-1', 'F1-2',... | LP first | 0.534860 |
| 1 | ['A1-1', 'A1-2', 'A1-3', 'C1', 'F1-1', 'F1-2',... | MILP first | 0.494870 |
| 2 | ['C2', 'A2', 'H1', 'H5'] | LP first | 0.528742 |
| 3 | ['C2', 'A2', 'H1', 'H5'] | MILP first | 0.533447 |
| 4 | ['C3', 'F4'] | LP first | 0.537164 |

In [152]:

```
df_results["Protein coverage"] = "all"
df_results_over_0["Protein coverage"] = "greater than 1e-7"
```

In [153]:

```
df_results_over_0 = df_results_over_0.rename({"saturation": "average"}, axis=1)
```

Remove global that the top coincides with the all coverage value when stacking

First sort so that the substraction is consistent.

In [154]:

```
df_results_over_0 = df_results_over_0.sort_values(["sample", "method"]).reset_index(drop=True)
df_results = df_results.sort_values(["sample", "method"]).reset_index(drop=True)
```

In [155]:

```
df_results_combined = pd.concat([df_results, df_results_over_0])
```

In [156]:

```
df_results_combined.to_csv(DATA / "average_saturation.tsv", sep="\t", index=False)
```

In [157]:

```
df_results_combined = pd.read_csv(DATA / "average_saturation.tsv", sep="\t")
```

In [158]:

```
df_results_combined = df_results_combined.rename({"sample": "Condition", "average": "Average saturation"}, axis=1)
```

In [159]:

```
df_results_combined["Condition"] = df_results_combined["Condition"].apply(lambda x: sample_to_condition[x])
```

In [160]:

```
df_results_combined["Protein coverage"] = df_results_combined["Protein coverage"].str.replace("greater than 1e-7", "used proteins")
```

In [161]:

```
import plotnine as p9
```

In [162]:

```
plot = (
    p9.ggplot(
        df_results_combined,
        p9.aes(
            x="Condition",
            y="Average saturation",
            fill="method",
            alpha="Protein coverage",
            color="Protein coverage",
            group="method",
        ),
    )
    + p9.geom_col(position="dodge")
    + p9.scale_alpha_discrete(range=[0.6, 0.5])
    + p9.scale_color_manual(values=["black", "white"])
    # default from plotly
    + p9.scale_fill_manual(values=["#636efa", "#ef553b"])
    + p9.theme_tufte()
    + p9.theme(plot_background=p9.element_rect(fill="white", color="white"), axis_text_x = p9.element_text(rotation=-45, ha="left", size=12), figure_size=(9,7), axis_title=p9.element_text(size=14))
)
plot
```

```
/home/georg/.virtualenvs/gecko-cplex/lib/python3.9/site-packages/plotnine/scales/scale_alpha.py:73: PlotnineWarning: Using alpha for a discrete variable is not advised.
```

Out[162]:

```
<Figure Size: (900 x 700)>
```

The top of each bar represents the average saturation over "used" enzymes while the bottom dent indicates the saturation accounting for all the enzymes with absolute proteomics added as a concentration.

In [163]:

```
plot.save("results_0448_avg_enzyme_saturation_and_zero_bw_additive_alpha.tiff", dpi=300)
```

```
/home/georg/.virtualenvs/gecko-cplex/lib/python3.9/site-packages/plotnine/ggplot.py:587: PlotnineWarning: Saving 9 x 7 in image.
/home/georg/.virtualenvs/gecko-cplex/lib/python3.9/site-packages/plotnine/ggplot.py:588: PlotnineWarning: Filename: results_0448_avg_enzyme_saturation_and_zero_bw_additive_alpha.tiff
```
