# Supplementary material for: Simultaneous application of enzyme and thermodynamic constraints to metabolic models using an updated Python implementation of GECKO
Source: Microbiol Spectr. 2023 Oct 16;11(6):e01705-23. doi: 10.1128/spectrum.01705-23 (PMC10783817; doi:10.1128/spectrum.01705-23)
Supplement: S3 file — Jupyter notebook to reproduce figure 4. [file spectrum.01705-23-s0003.html]

fva\_comparisons


# Figure 4 of geckopy 3.0 manuscript¶

Adapted from pytfa tutorials.

##### Index¶

- Flux Variability Analysis
  - Plain FVA.
  - Thermo FVA.
  - Thermo concentrations FVA.
  - Proteomics FVA.
  - Thermo protein concentrations FVA.
  - Protein pool FVA.
- Plotting

In [1]:

```
# import os
import errno
import logging
import re
from math import isnan, log
from os.path import join, pardir
from typing import List
import cobra
import geckopy
import matplotlib.pyplot as plt
import numpy as np
import pandas as pd
import pytfa
from cobra.flux_analysis.variability import flux_variability_analysis
from geckopy.experimental import from_copy_number
from geckopy.experimental.molecular_weights import extract_proteins
from geckopy.experimental.relaxation import (
    Objective_rule,
    apply_proteomics_elastic_relaxation,
    apply_proteomics_relaxation,
    elastic_upper_relaxation,
    get_upper_relaxation,
    relax_proteomics_greedy,
)
from geckopy.integration import relax_thermo_proteins
from geckopy.integration.pytfa import (
    adapt_gecko_to_thermo,
    get_thermo_coverage,
    get_thermo_reactions,
    translate_model_mnx_to_seed,
)
from pytfa.analysis import variability_analysis
from pytfa.io import load_thermoDB
from pytfa.io.plotting import plot_fva_tva_comparison
from pytfa.optim.constraints import (
    BackwardDeltaGCoupling,
    BackwardDirectionCoupling,
    DisplacementCoupling,
    ForwardDeltaGCoupling,
    ForwardDirectionCoupling,
    MetaboliteConstraint,
    NegativeDeltaG,
    ReactionConstraint,
    SimultaneousUse,
)
from pytfa.optim.relaxation import relax_dgo
from pytfa.optim.utils import get_solution_value_for_variables
from pytfa.optim.variables import (
    BackwardUseVariable,
    DeltaG,
    DeltaGstd,
    ForwardUseVariable,
    LogConcentration,
)
```

In [2]:

```
CPLEX = "optlang-cplex"
GUROBI = "optlang-gurobi"
GLPK = "optlang-glpk"
ROOT = pardir
DATA = join(ROOT, "data")
```

### Load the model¶

Load the enzyme constraint model and transform it to a thermo model (taking into account the proteins).

In [3]:

```
ec_model = geckopy.io.read_sbml_ec_model(
    join(DATA, "ec_coli_core.xml"), hardcoded_rev_reactions=False
)
# ec_model = geckopy.io.read_sbml_ec_model(join(DATA, "eciML1515_seed.xml"))
```

In [4]:

```
ec_model.solver = "cplex"
```

In [5]:

```
ec_model.slim_optimize()
# ec_model.reactions.EX_glc__D_e.bounds = ec_model.reactions.EX_glc__D_e.flux, ec_model.reactions.EX_glc__D_e.flux
```

Out[5]:

```
0.8739215069684302
```

In [6]:

```
## Transfrom it into thermo model
def create_thermo(ec_model):
    thermodb = load_thermoDB(join(DATA, "thermo_data.thermodb"))
    compartment_data = pytfa.io.read_compartment_data(
        join(DATA, "compartment_data.json")
    )
    #     translate_model_mnx_to_seed(
    #         ec_model, thermodb, join(DATA, "chem_xref_seedset.tsv")
    #     )
    tmodel = adapt_gecko_to_thermo(ec_model, thermodb, compartment_data, solver=CPLEX)
    return tmodel


# Info on the cobra_model
tmodel = create_thermo(ec_model)
tmodel.print_info()
```

```
2023-08-09 14:23:36,559 - thermomodel_ - INFO - # Model initialized with units kcal/mol and temperature 298.15 K
2023-08-09 14:23:36,569 - thermomodel_ - INFO - # Model preparation starting...
2023-08-09 14:23:36,627 - thermomodel_ - INFO - # Model preparation done.
2023-08-09 14:23:36,662 - thermomodel_ - INFO - # Model conversion starting...
2023-08-09 14:23:38,061 - thermomodel_ - INFO - # Model conversion done.
2023-08-09 14:23:38,062 - thermomodel_ - INFO - # Updating cobra_model variables...
2023-08-09 14:23:38,070 - thermomodel_ - INFO - # cobra_model variables are up-to-date
```

```
                value
key                  
name                 
description          
num constraints   631
num variables     763
num metabolites    72
num reactions      95
                              value
key                                
num metabolites(thermo)  127.000000
num reactions(thermo)     73.000000
pct metabolites(thermo)  176.388889
pct reactions(thermo)     76.842105
```

Check that the model works.

In [7]:

```
tmodel.slim_optimize()
```

Out[7]:

```
0.3742298749331099
```

### Flux variability analysis¶

Calculate variability analysis on all continuous variables

#### Plain FVA¶

In [8]:

```
logging.basicConfig(filename="figures_core.log", level=logging.DEBUG)
```

In [9]:

```
ec_model.logger = logging.getLogger(__name__)
fva_fluxes = variability_analysis(ec_model.copy(), kind="reaction")
```

```
minimizing: 100%|███████████████████████████████████████████| 95/95 [00:00<00:00, 330.85it/s]
maximizing: 100%|███████████████████████████████████████████| 95/95 [00:00<00:00, 510.58it/s]
```

The solver is using all available processors, so we set `n_proc` to 1 to avoid threading overhead.

#### Thermo FVA¶

In [10]:

```
tva_fluxes = variability_analysis(tmodel, kind="reaction")
```

```
2023-08-09 14:23:39,097 - thermomodel_ - INFO - Beginning variability analysis for variable of type reaction
minimizing: 100%|████████████████████████████████████████████| 95/95 [00:09<00:00, 10.53it/s]
maximizing: 100%|████████████████████████████████████████████| 95/95 [00:15<00:00,  6.13it/s]
```

Save the results just in case.

In [11]:

```
fva_fluxes.to_csv("fva_fluxes_core.tsv", sep="\t", index_label="reaction")
tva_fluxes.to_csv("tva_fluxes_core.tsv", sep="\t", index_label="reaction")
```

#### Thermo concentrations FVA¶

##### Now, the same with specific concentration data.¶

In [12]:

```
# Add more specific concentration data
def apply_concentration_bound(met, lb, ub):
    the_conc_var = tmodel.log_concentration.get_by_id(met)
    # Do not forget the variables in the model are logs !
    the_conc_var.ub = log(ub)
    the_conc_var.lb = log(lb)
```

In [13]:

```
tmodel = create_thermo(ec_model)
```

```
2023-08-09 14:24:04,362 - thermomodel_ - INFO - # Model initialized with units kcal/mol and temperature 298.15 K
2023-08-09 14:24:04,368 - thermomodel_ - INFO - # Model preparation starting...
2023-08-09 14:24:04,417 - thermomodel_ - INFO - # Model preparation done.
2023-08-09 14:24:04,447 - thermomodel_ - INFO - # Model conversion starting...
2023-08-09 14:24:05,699 - thermomodel_ - INFO - # Model conversion done.
2023-08-09 14:24:05,700 - thermomodel_ - INFO - # Updating cobra_model variables...
2023-08-09 14:24:05,705 - thermomodel_ - INFO - # cobra_model variables are up-to-date
```

In [14]:

```
apply_concentration_bound("atp_c", lb=1e-4, ub=5e-4)
apply_concentration_bound("adp_c", lb=4e-4, ub=7e-4)
apply_concentration_bound("amp_c", lb=2e-5, ub=3e-5)

tmodel.optimize()
# Perform variability analysis again
tva_fluxes_lc = variability_analysis(tmodel, kind="reactions")
```

```
2023-08-09 14:24:06,036 - thermomodel_ - INFO - Beginning variability analysis for variable of type reactions
minimizing: 100%|████████████████████████████████████████████| 95/95 [00:08<00:00, 11.32it/s]
maximizing: 100%|████████████████████████████████████████████| 95/95 [00:14<00:00,  6.75it/s]
```

In [15]:

```
tva_fluxes_lc.to_csv("tva_fluxes_lc_core.tsv", sep="\t", index_label="reaction")
```

#### Proteomics FVA¶

In [16]:

```
raw_proteomics = pd.read_csv(join(DATA, "ecoli_proteomics_schmidt2016S5.tsv"))
# the proteomics in the model have a "prot_" prefix
raw_proteomics.uniprot = raw_proteomics.uniprot.apply(lambda x: f"prot_{x}")
ec_model_constrained = from_copy_number(
    ec_model.copy(),
    index=raw_proteomics["uniprot"],
    cell_copies=raw_proteomics["copies_per_cell"],
    stdev=raw_proteomics["stdev"],
    vol=2.3,
    dens=1.105e-12,
    water=0.3,
)
```

In [17]:

```
prots_with_concentration = [
    prot.id
    for prot in ec_model_constrained.proteins
    if prot.concentration is not None and not isnan(prot.concentration)
]
```

In [18]:

```
ec_model_constrained.reactions.BIOMASS_Ecoli_core_w_GAM.lower_bound = (
    tmodel.slim_optimize()
)
iis_lpobj = elastic_upper_relaxation(
    ec_model_constrained,
    prots_with_concentration,
    Objective_rule.MIN_ELASTIC_SUM_OBJECTIVE,
)
iis_lp = elastic_upper_relaxation(
    ec_model_constrained, prots_with_concentration, Objective_rule.MIN_ELASTIC_SUM
)
iis_milp = elastic_upper_relaxation(
    ec_model_constrained, prots_with_concentration, Objective_rule.MIN_MILP_COUNT
)
```

All rules returned the same proteins.

In [19]:

```
ec_model_constrained.reactions.BIOMASS_Ecoli_core_w_GAM.lower_bound = 0.0
```

In [20]:

```
len(iis_milp)
```

Out[20]:

```
20
```

In [21]:

```
new_growth_rate, prots_relaxed = relax_proteomics_greedy(
    ec_model_constrained, minimal_growth=tmodel.slim_optimize(), protein_set=iis_milp
)
```

```
/home/georg/.virtualenvs/gecko-cplex/lib/python3.9/site-packages/cobra/util/solver.py:554: UserWarning: Solver status is 'infeasible'.
  warn(f"Solver status is '{status}'.", UserWarning)
/home/georg/.virtualenvs/gecko-cplex/lib/python3.9/site-packages/cobra/util/solver.py:554: UserWarning: Solver status is 'infeasible'.
  warn(f"Solver status is '{status}'.", UserWarning)
/home/georg/.virtualenvs/gecko-cplex/lib/python3.9/site-packages/geckopy/experimental/relaxation.py:282: FutureWarning: Passing a set as an indexer is deprecated and will raise in a future version. Use a list instead.
  return shadow_pr[protein_set].sort_values()[:top]
/home/georg/.virtualenvs/gecko-cplex/lib/python3.9/site-packages/cobra/util/solver.py:554: UserWarning: Solver status is 'infeasible'.
  warn(f"Solver status is '{status}'.", UserWarning)
/home/georg/.virtualenvs/gecko-cplex/lib/python3.9/site-packages/cobra/util/solver.py:554: UserWarning: Solver status is 'infeasible'.
  warn(f"Solver status is '{status}'.", UserWarning)
/home/georg/.virtualenvs/gecko-cplex/lib/python3.9/site-packages/geckopy/experimental/relaxation.py:282: FutureWarning: Passing a set as an indexer is deprecated and will raise in a future version. Use a list instead.
  return shadow_pr[protein_set].sort_values()[:top]
/home/georg/.virtualenvs/gecko-cplex/lib/python3.9/site-packages/cobra/util/solver.py:554: UserWarning: Solver status is 'infeasible'.
  warn(f"Solver status is '{status}'.", UserWarning)
/home/georg/.virtualenvs/gecko-cplex/lib/python3.9/site-packages/cobra/util/solver.py:554: UserWarning: Solver status is 'infeasible'.
  warn(f"Solver status is '{status}'.", UserWarning)
/home/georg/.virtualenvs/gecko-cplex/lib/python3.9/site-packages/geckopy/experimental/relaxation.py:282: FutureWarning: Passing a set as an indexer is deprecated and will raise in a future version. Use a list instead.
  return shadow_pr[protein_set].sort_values()[:top]
/home/georg/.virtualenvs/gecko-cplex/lib/python3.9/site-packages/cobra/util/solver.py:554: UserWarning: Solver status is 'infeasible'.
  warn(f"Solver status is '{status}'.", UserWarning)
/home/georg/.virtualenvs/gecko-cplex/lib/python3.9/site-packages/cobra/util/solver.py:554: UserWarning: Solver status is 'infeasible'.
  warn(f"Solver status is '{status}'.", UserWarning)
/home/georg/.virtualenvs/gecko-cplex/lib/python3.9/site-packages/geckopy/experimental/relaxation.py:282: FutureWarning: Passing a set as an indexer is deprecated and will raise in a future version. Use a list instead.
  return shadow_pr[protein_set].sort_values()[:top]
/home/georg/.virtualenvs/gecko-cplex/lib/python3.9/site-packages/cobra/util/solver.py:554: UserWarning: Solver status is 'infeasible'.
  warn(f"Solver status is '{status}'.", UserWarning)
/home/georg/.virtualenvs/gecko-cplex/lib/python3.9/site-packages/cobra/util/solver.py:554: UserWarning: Solver status is 'infeasible'.
  warn(f"Solver status is '{status}'.", UserWarning)
/home/georg/.virtualenvs/gecko-cplex/lib/python3.9/site-packages/geckopy/experimental/relaxation.py:282: FutureWarning: Passing a set as an indexer is deprecated and will raise in a future version. Use a list instead.
  return shadow_pr[protein_set].sort_values()[:top]
/home/georg/.virtualenvs/gecko-cplex/lib/python3.9/site-packages/cobra/util/solver.py:554: UserWarning: Solver status is 'infeasible'.
  warn(f"Solver status is '{status}'.", UserWarning)
/home/georg/.virtualenvs/gecko-cplex/lib/python3.9/site-packages/cobra/util/solver.py:554: UserWarning: Solver status is 'infeasible'.
  warn(f"Solver status is '{status}'.", UserWarning)
/home/georg/.virtualenvs/gecko-cplex/lib/python3.9/site-packages/geckopy/experimental/relaxation.py:282: FutureWarning: Passing a set as an indexer is deprecated and will raise in a future version. Use a list instead.
  return shadow_pr[protein_set].sort_values()[:top]
/home/georg/.virtualenvs/gecko-cplex/lib/python3.9/site-packages/cobra/util/solver.py:554: UserWarning: Solver status is 'infeasible'.
  warn(f"Solver status is '{status}'.", UserWarning)
/home/georg/.virtualenvs/gecko-cplex/lib/python3.9/site-packages/cobra/util/solver.py:554: UserWarning: Solver status is 'infeasible'.
  warn(f"Solver status is '{status}'.", UserWarning)
/home/georg/.virtualenvs/gecko-cplex/lib/python3.9/site-packages/geckopy/experimental/relaxation.py:282: FutureWarning: Passing a set as an indexer is deprecated and will raise in a future version. Use a list instead.
  return shadow_pr[protein_set].sort_values()[:top]
/home/georg/.virtualenvs/gecko-cplex/lib/python3.9/site-packages/cobra/util/solver.py:554: UserWarning: Solver status is 'infeasible'.
  warn(f"Solver status is '{status}'.", UserWarning)
/home/georg/.virtualenvs/gecko-cplex/lib/python3.9/site-packages/cobra/util/solver.py:554: UserWarning: Solver status is 'infeasible'.
  warn(f"Solver status is '{status}'.", UserWarning)
/home/georg/.virtualenvs/gecko-cplex/lib/python3.9/site-packages/geckopy/experimental/relaxation.py:282: FutureWarning: Passing a set as an indexer is deprecated and will raise in a future version. Use a list instead.
  return shadow_pr[protein_set].sort_values()[:top]
/home/georg/.virtualenvs/gecko-cplex/lib/python3.9/site-packages/cobra/util/solver.py:554: UserWarning: Solver status is 'infeasible'.
  warn(f"Solver status is '{status}'.", UserWarning)
/home/georg/.virtualenvs/gecko-cplex/lib/python3.9/site-packages/cobra/util/solver.py:554: UserWarning: Solver status is 'infeasible'.
  warn(f"Solver status is '{status}'.", UserWarning)
/home/georg/.virtualenvs/gecko-cplex/lib/python3.9/site-packages/geckopy/experimental/relaxation.py:282: FutureWarning: Passing a set as an indexer is deprecated and will raise in a future version. Use a list instead.
  return shadow_pr[protein_set].sort_values()[:top]
/home/georg/.virtualenvs/gecko-cplex/lib/python3.9/site-packages/cobra/util/solver.py:554: UserWarning: Solver status is 'infeasible'.
  warn(f"Solver status is '{status}'.", UserWarning)
/home/georg/.virtualenvs/gecko-cplex/lib/python3.9/site-packages/cobra/util/solver.py:554: UserWarning: Solver status is 'infeasible'.
  warn(f"Solver status is '{status}'.", UserWarning)
/home/georg/.virtualenvs/gecko-cplex/lib/python3.9/site-packages/geckopy/experimental/relaxation.py:282: FutureWarning: Passing a set as an indexer is deprecated and will raise in a future version. Use a list instead.
  return shadow_pr[protein_set].sort_values()[:top]
/home/georg/.virtualenvs/gecko-cplex/lib/python3.9/site-packages/cobra/util/solver.py:554: UserWarning: Solver status is 'infeasible'.
  warn(f"Solver status is '{status}'.", UserWarning)
/home/georg/.virtualenvs/gecko-cplex/lib/python3.9/site-packages/cobra/util/solver.py:554: UserWarning: Solver status is 'infeasible'.
  warn(f"Solver status is '{status}'.", UserWarning)
/home/georg/.virtualenvs/gecko-cplex/lib/python3.9/site-packages/geckopy/experimental/relaxation.py:282: FutureWarning: Passing a set as an indexer is deprecated and will raise in a future version. Use a list instead.
  return shadow_pr[protein_set].sort_values()[:top]
/home/georg/.virtualenvs/gecko-cplex/lib/python3.9/site-packages/cobra/util/solver.py:554: UserWarning: Solver status is 'infeasible'.
  warn(f"Solver status is '{status}'.", UserWarning)
/home/georg/.virtualenvs/gecko-cplex/lib/python3.9/site-packages/cobra/util/solver.py:554: UserWarning: Solver status is 'infeasible'.
  warn(f"Solver status is '{status}'.", UserWarning)
/home/georg/.virtualenvs/gecko-cplex/lib/python3.9/site-packages/geckopy/experimental/relaxation.py:282: FutureWarning: Passing a set as an indexer is deprecated and will raise in a future version. Use a list instead.
  return shadow_pr[protein_set].sort_values()[:top]
/home/georg/.virtualenvs/gecko-cplex/lib/python3.9/site-packages/cobra/util/solver.py:554: UserWarning: Solver status is 'infeasible'.
  warn(f"Solver status is '{status}'.", UserWarning)
/home/georg/.virtualenvs/gecko-cplex/lib/python3.9/site-packages/cobra/util/solver.py:554: UserWarning: Solver status is 'infeasible'.
  warn(f"Solver status is '{status}'.", UserWarning)
/home/georg/.virtualenvs/gecko-cplex/lib/python3.9/site-packages/geckopy/experimental/relaxation.py:282: FutureWarning: Passing a set as an indexer is deprecated and will raise in a future version. Use a list instead.
  return shadow_pr[protein_set].sort_values()[:top]
/home/georg/.virtualenvs/gecko-cplex/lib/python3.9/site-packages/cobra/util/solver.py:554: UserWarning: Solver status is 'infeasible'.
  warn(f"Solver status is '{status}'.", UserWarning)
/home/georg/.virtualenvs/gecko-cplex/lib/python3.9/site-packages/cobra/util/solver.py:554: UserWarning: Solver status is 'infeasible'.
  warn(f"Solver status is '{status}'.", UserWarning)
/home/georg/.virtualenvs/gecko-cplex/lib/python3.9/site-packages/geckopy/experimental/relaxation.py:282: FutureWarning: Passing a set as an indexer is deprecated and will raise in a future version. Use a list instead.
  return shadow_pr[protein_set].sort_values()[:top]
/home/georg/.virtualenvs/gecko-cplex/lib/python3.9/site-packages/cobra/util/solver.py:554: UserWarning: Solver status is 'infeasible'.
  warn(f"Solver status is '{status}'.", UserWarning)
/home/georg/.virtualenvs/gecko-cplex/lib/python3.9/site-packages/cobra/util/solver.py:554: UserWarning: Solver status is 'infeasible'.
  warn(f"Solver status is '{status}'.", UserWarning)
/home/georg/.virtualenvs/gecko-cplex/lib/python3.9/site-packages/geckopy/experimental/relaxation.py:282: FutureWarning: Passing a set as an indexer is deprecated and will raise in a future version. Use a list instead.
  return shadow_pr[protein_set].sort_values()[:top]
/home/georg/.virtualenvs/gecko-cplex/lib/python3.9/site-packages/cobra/util/solver.py:554: UserWarning: Solver status is 'infeasible'.
  warn(f"Solver status is '{status}'.", UserWarning)
/home/georg/.virtualenvs/gecko-cplex/lib/python3.9/site-packages/cobra/util/solver.py:554: UserWarning: Solver status is 'infeasible'.
  warn(f"Solver status is '{status}'.", UserWarning)
/home/georg/.virtualenvs/gecko-cplex/lib/python3.9/site-packages/geckopy/experimental/relaxation.py:282: FutureWarning: Passing a set as an indexer is deprecated and will raise in a future version. Use a list instead.
  return shadow_pr[protein_set].sort_values()[:top]
/home/georg/.virtualenvs/gecko-cplex/lib/python3.9/site-packages/cobra/util/solver.py:554: UserWarning: Solver status is 'infeasible'.
  warn(f"Solver status is '{status}'.", UserWarning)
/home/georg/.virtualenvs/gecko-cplex/lib/python3.9/site-packages/cobra/util/solver.py:554: UserWarning: Solver status is 'infeasible'.
  warn(f"Solver status is '{status}'.", UserWarning)
/home/georg/.virtualenvs/gecko-cplex/lib/python3.9/site-packages/geckopy/experimental/relaxation.py:282: FutureWarning: Passing a set as an indexer is deprecated and will raise in a future version. Use a list instead.
  return shadow_pr[protein_set].sort_values()[:top]
/home/georg/.virtualenvs/gecko-cplex/lib/python3.9/site-packages/cobra/util/solver.py:554: UserWarning: Solver status is 'infeasible'.
  warn(f"Solver status is '{status}'.", UserWarning)
/home/georg/.virtualenvs/gecko-cplex/lib/python3.9/site-packages/cobra/util/solver.py:554: UserWarning: Solver status is 'infeasible'.
  warn(f"Solver status is '{status}'.", UserWarning)
/home/georg/.virtualenvs/gecko-cplex/lib/python3.9/site-packages/geckopy/experimental/relaxation.py:282: FutureWarning: Passing a set as an indexer is deprecated and will raise in a future version. Use a list instead.
  return shadow_pr[protein_set].sort_values()[:top]
/home/georg/.virtualenvs/gecko-cplex/lib/python3.9/site-packages/cobra/util/solver.py:554: UserWarning: Solver status is 'infeasible'.
  warn(f"Solver status is '{status}'.", UserWarning)
/home/georg/.virtualenvs/gecko-cplex/lib/python3.9/site-packages/cobra/util/solver.py:554: UserWarning: Solver status is 'infeasible'.
  warn(f"Solver status is '{status}'.", UserWarning)
/home/georg/.virtualenvs/gecko-cplex/lib/python3.9/site-packages/geckopy/experimental/relaxation.py:282: FutureWarning: Passing a set as an indexer is deprecated and will raise in a future version. Use a list instead.
  return shadow_pr[protein_set].sort_values()[:top]
/home/georg/.virtualenvs/gecko-cplex/lib/python3.9/site-packages/cobra/util/solver.py:554: UserWarning: Solver status is 'infeasible'.
  warn(f"Solver status is '{status}'.", UserWarning)
/home/georg/.virtualenvs/gecko-cplex/lib/python3.9/site-packages/cobra/util/solver.py:554: UserWarning: Solver status is 'infeasible'.
  warn(f"Solver status is '{status}'.", UserWarning)
/home/georg/.virtualenvs/gecko-cplex/lib/python3.9/site-packages/geckopy/experimental/relaxation.py:282: FutureWarning: Passing a set as an indexer is deprecated and will raise in a future version. Use a list instead.
  return shadow_pr[protein_set].sort_values()[:top]
```

In [22]:

```
tva_fluxes_prot = variability_analysis(ec_model_constrained, kind="reaction")
```

```
minimizing: 100%|███████████████████████████████████████████| 95/95 [00:00<00:00, 367.69it/s]
maximizing: 100%|███████████████████████████████████████████| 95/95 [00:00<00:00, 634.02it/s]
```

In [23]:

```
tva_fluxes_prot.to_csv(
    "tva_fluxes_just_prot_core.tsv", sep="\t", index_label="reaction"
)
```

#### Thermo concentration proteomics FVA¶

In [24]:

```
raw_proteomics = pd.read_csv(join(DATA, "ecoli_proteomics_schmidt2016S5.tsv"))
# the proteomics in the model have a "prot_" prefix
raw_proteomics.uniprot = raw_proteomics.uniprot.apply(lambda x: f"prot_{x}")
ec_model_constrained = from_copy_number(
    ec_model.copy(),
    index=raw_proteomics["uniprot"],
    cell_copies=raw_proteomics["copies_per_cell"],
    stdev=raw_proteomics["stdev"],
    vol=2.3,
    dens=1.105e-12,
    water=0.3,
)
tmodel_prot = create_thermo(ec_model_constrained)
```

```
2023-08-09 14:24:32,830 - thermomodel_ - INFO - # Model initialized with units kcal/mol and temperature 298.15 K
2023-08-09 14:24:32,840 - thermomodel_ - INFO - # Model preparation starting...
2023-08-09 14:24:32,895 - thermomodel_ - INFO - # Model preparation done.
2023-08-09 14:24:32,945 - thermomodel_ - INFO - # Model conversion starting...
2023-08-09 14:24:34,315 - thermomodel_ - INFO - # Model conversion done.
2023-08-09 14:24:34,316 - thermomodel_ - INFO - # Updating cobra_model variables...
2023-08-09 14:24:34,321 - thermomodel_ - INFO - # cobra_model variables are up-to-date
```

In [25]:

```
tmodel_prot.slim_optimize()
```

Out[25]:

```
nan
```

In [26]:

```
tmodel_prot.reactions.BIOMASS_Ecoli_core_w_GAM.lower_bound = tmodel.slim_optimize()
iis_milp = relax_thermo_proteins(
    tmodel_prot, prots_with_concentration, Objective_rule.MIN_MILP_COUNT
)
```

```
adding thermo slacks: 100%|█████████████████████████████████| 73/73 [00:00<00:00, 103.51it/s]
adding protein slacks: 100%|████████████████████████████████| 49/49 [00:00<00:00, 130.58it/s]
```

In [27]:

```
len(iis_milp[0])
```

Out[27]:

```
22
```

In [28]:

```
raw_proteomics = pd.read_csv(join(DATA, "ecoli_proteomics_schmidt2016S5.tsv"))
# the proteomics in the model have a "prot_" prefix
raw_proteomics.uniprot = raw_proteomics.uniprot.apply(lambda x: f"prot_{x}")
ec_model_constrained = from_copy_number(
    ec_model.copy(),
    index=raw_proteomics["uniprot"],
    cell_copies=raw_proteomics["copies_per_cell"],
    stdev=raw_proteomics["stdev"],
    vol=2.3,
    dens=1.105e-12,
    water=0.3,
)
tmodel_prot = create_thermo(ec_model_constrained)
for prot in iis_milp[0]:
    tmodel_prot.proteins.get_by_id(prot).concentration = None
```

```
2023-08-09 14:24:37,388 - thermomodel_ - INFO - # Model initialized with units kcal/mol and temperature 298.15 K
2023-08-09 14:24:37,398 - thermomodel_ - INFO - # Model preparation starting...
2023-08-09 14:24:37,482 - thermomodel_ - INFO - # Model preparation done.
2023-08-09 14:24:37,534 - thermomodel_ - INFO - # Model conversion starting...
2023-08-09 14:24:39,018 - thermomodel_ - INFO - # Model conversion done.
2023-08-09 14:24:39,019 - thermomodel_ - INFO - # Updating cobra_model variables...
2023-08-09 14:24:39,024 - thermomodel_ - INFO - # cobra_model variables are up-to-date
```

In [29]:

```
tmodel_prot.optimize()
```

Out[29]:

***Optimal* solution with objective value 0.374**  

|  | fluxes | reduced\_costs |
| --- | --- | --- |
| PFK | 9.627754 | None |
| PFL | 0.000000 | None |
| PGI | 9.923283 | None |
| PGK | -19.005185 | None |
| PGL | 0.000000 | None |
| ... | ... | ... |
| NADH16 | 30.034558 | None |
| NADTRHD | 64.916286 | None |
| NH4t | 2.040601 | None |
| O2t | 15.017279 | None |
| PDH | 16.118563 | None |

95 rows × 2 columns

In [30]:

```
# Perform variability analysis again
tva_fluxes_prot = variability_analysis(tmodel_prot, kind="reactions")
```

```
2023-08-09 14:24:39,426 - thermomodel_ - INFO - Beginning variability analysis for variable of type reactions
minimizing: 100%|████████████████████████████████████████████| 95/95 [00:09<00:00,  9.55it/s]
maximizing: 100%|████████████████████████████████████████████| 95/95 [00:06<00:00, 14.19it/s]
```

In [31]:

```
tva_fluxes_prot.to_csv("tva_fluxes_prot_core.tsv", sep="\t", index_label="reaction")
```

#### Protein pool FVA¶

In [32]:

```
pool_model = geckopy.io.read_sbml_ec_model(
    join(DATA, "ec_coli_core.xml"), hardcoded_rev_reactions=False
)
pool_model.solver = "cplex"
pool_model.reactions.EX_glc__D_e.bounds = (
    ec_model.reactions.EX_glc__D_e.flux,
    ec_model.reactions.EX_glc__D_e.flux,
)
pool_model.slim_optimize()
```

Out[32]:

```
0.8739215069684301
```

In [35]:

```
df = extract_proteins(pool_model)
```

In [36]:

```
for row in df.itertuples():
    pool_model.proteins.get_by_id(row[2]).mw = row[3]
```

In [37]:

```
pool_model.constrain_pool(
    0.448,
    0.6,
    1.0,
)
```

In [38]:

```
pool_model.slim_optimize()
```

Out[38]:

```
0.8739215069684301
```

We take the smaller set.

In [39]:

```
pool_model.reactions.BIOMASS_Ecoli_core_w_GAM.lower_bound = 0
```

In [40]:

```
pool_model.slim_optimize()
```

Out[40]:

```
0.8739215069684301
```

In [41]:

```
pool_model.logger = logging.getLogger(__name__)
tva_fluxes_pool = variability_analysis(pool_model)
```

```
minimizing: 100%|███████████████████████████████████████████| 96/96 [00:00<00:00, 412.34it/s]
maximizing: 100%|███████████████████████████████████████████| 96/96 [00:00<00:00, 491.56it/s]
```

In [42]:

```
tva_fluxes_pool.to_csv("tva_fluxes_pool_core.tsv", sep="\t", index_label="reaction")
```

### Plotting¶

In [5]:

```
def plot_stacked_negative(
    dfs=List[pd.DataFrame],
    labels=List[str],
    colors=List[str],
    widths=List[float],
    width_col=0.12,
):
    """Plot stacked negative barplot."""
    n_rows = dfs[0].shape[0]
    ind = np.arange(n_rows)
    fig, ax = plt.subplots(figsize=(18, 6))

    for df, label, color, width in zip(dfs, labels, colors, widths):
        p1 = ax.bar(ind + width, df.maximum, width_col, label=label, color=color)
        p2 = ax.bar(ind + width, df.minimum, width_col, color=color)

    ax.axhline(0, color="#221c22", linewidth=0.8, linestyle="--")
    ax.set_ylabel("Fluxes")
    ax.set_xticks(ind)
    ax.set_xticklabels(dfs[0].index, rotation=-40)
    ax.legend()


def apply_rev(reac_id, df, model):
    direction = "maximum"
    rev_id = re.sub(REV_PATTERN, r"_REV\1", reac_id)
    try:
        rev_id = model.reactions.get_by_id(rev_id).id
    except KeyError:
        rev_id = reac_id
        direction = "minimum"
    return -df.loc[rev_id, direction]


def apply_df_rev(df, model):
    df["minimum"] = [apply_rev(reac_id, df, ec_model) for reac_id in df.index]
```

Load previously generated output.

In [6]:

```
fva_fluxes = pd.read_csv("fva_fluxes_core.tsv", sep="\t", index_col="reaction").assign(
    method="FBA"
)
tva_fluxes = pd.read_csv("tva_fluxes_core.tsv", sep="\t", index_col="reaction").assign(
    method="Thermo"
)
tva_fluxes_lc = pd.read_csv(
    "tva_fluxes_lc_core.tsv", sep="\t", index_col="reaction"
).assign(method="Thermo + Metabolomics")
tva_fluxes_prot = pd.read_csv(
    "tva_fluxes_prot_core.tsv", sep="\t", index_col="reaction"
).assign(method="Thermo + Proteomics")
tva_fluxes_just_prot = pd.read_csv(
    "tva_fluxes_just_prot_core.tsv", sep="\t", index_col="reaction"
).assign(method="Proteomics")
tva_fluxes_pool = pd.read_csv(
    "tva_fluxes_pool_core.tsv", sep="\t", index_col="reaction"
).assign(method="Pool constraint")
```

In [7]:

```
dfs = (
    fva_fluxes,
    tva_fluxes,
    tva_fluxes_lc,
    tva_fluxes_prot,
    tva_fluxes_pool,
    tva_fluxes_just_prot,
)
```

In [8]:

```
dfsub = pd.concat(
    [
        fva_fluxes,
        tva_fluxes.rename({"minimum": "min_prot", "maximum": "max_prot"}, axis=1),
    ],
    axis=1,
)
reacs_to_plot = (
    dfsub.apply(lambda s: s.maximum - s.minimum + s.min_prot - s.max_prot, axis=1)
    .sort_values(ascending=False)[:20]
    .index
)
```

In [47]:

```
dfsub = pd.concat(
    [
        fva_fluxes,
        tva_fluxes_prot.rename({"minimum": "min_prot", "maximum": "max_prot"}, axis=1),
    ],
    axis=1,
)
reacs_to_plot.append(
    dfsub.apply(lambda s: s.minimum - s.min_prot, axis=1).sort_values()[:10].index
)
```

Out[47]:

```
Index(['GLUDy', 'GLUSy', 'PPS', 'GLNS', 'GLUN', 'PYK', 'ADK1', 'ME2', 'ME1',
       'MDH', 'GND', 'G6PDH2r', 'PGL', 'PGI', 'RPE', 'TKT2', 'TKT1', 'TALA',
       'RPI', 'NADH16', 'FORt', 'MDH', 'PGI', 'CO2t', 'EX_o2_e', 'H2Ot',
       'D_LACt2', 'LDH_D', 'RPI', 'FUM'],
      dtype='object', name='reaction')
```

In [9]:

```
reacs_to_plot = [
    "ATPS4r",
    "NADTRHD",
    "PPCK",
    "ADK1",
    "FBA",
    "G6PDH2r",
    "PFK",
    "PPS",
    "PYK",
    "TPI",
]
```

Figure 5 BEC of Sánchez et al., 2017.

In [10]:

```
df_plot = pd.concat(
    [
        abs(df.maximum - df.minimum)
        .apply(lambda x: x if x < 2000 else 2000)
        .to_frame()
        .assign(method=df.iloc[0, 2])
        .rename({0: "Flux"}, axis=1)
        for df in dfs
    ]
)
```

In [12]:

```
df_plot
```

Out[12]:

|  | Flux | method |
| --- | --- | --- |
| reaction |  |  |
| PFK | 176.610000 | FBA |
| PFL | 40.000000 | FBA |
| PGI | 60.000000 | FBA |
| PGK | 20.000000 | FBA |
| PGL | 60.000000 | FBA |
| ... | ... | ... |
| NADH16 | 47.052604 | Proteomics |
| NADTRHD | 0.202139 | Proteomics |
| NH4t | 2.957808 | Proteomics |
| O2t | 27.427014 | Proteomics |
| PDH | 20.196188 | Proteomics |

571 rows × 2 columns

In [11]:

```
abs(tva_fluxes_pool.maximum - tva_fluxes_pool.minimum).apply(
    lambda x: x if x < 2000 else 2000
).max()
```

Out[11]:

```
1000.0
```

In [13]:

```
import plotly.express as px
```

In [14]:

```
fig = px.histogram(
    df_plot[df_plot.Flux.abs() > 1e-7],
    x="Flux",
    color="method",
    cumulative=True,
    nbins=1000,
    marginal="box",
    barmode="overlay",
    opacity=0.1,
    width=800,
    height=600,
)
fig.update_layout(
    legend=dict(
        x=0.7,
        y=0.05,
        traceorder="normal",
        font=dict(family="sans-serif", size=12, color="black"),
    )
)
```

In [15]:

```
fig = px.box(
    df_plot[df_plot.Flux.abs() > 1e-7],
    x="Flux",
    y="method",
    color="method",
    log_x=True,
    template="plotly_white",
    width=800,
    height=600,
)
fig.update_layout(showlegend=False, yaxis={"tickangle": -45})

fig.update_xaxes(title_font={"size": 20}, tickfont=dict(size=14))
fig.update_yaxes(tickfont=dict(size=16), title=None)
fig
```

In [19]:

```
fig.write_image("/home/georg/Downloads/fva_comp.pdf")
fig.write_image("/home/georg/Downloads/fva_comp.svg")
```

In [20]:

```
df_plot_b = pd.concat(dfs)
df_plot_b["Reaction"] = df_plot.index
# df_plot_b.loc[(df_plot_b.minimum - df_plot_b.maximum).abs() < 1e-4, "minimum"] = 0.
df_plot_b = df_plot_b.melt(["method", "Reaction"], var_name="Sense", value_name="Flux")
df_plot_b
```

Out[20]:

|  | method | Reaction | Sense | Flux |
| --- | --- | --- | --- | --- |
| 0 | FBA | PFK | minimum | 0.000000 |
| 1 | FBA | PFL | minimum | 0.000000 |
| 2 | FBA | PGI | minimum | -50.000000 |
| 3 | FBA | PGK | minimum | -20.000000 |
| 4 | FBA | PGL | minimum | 0.000000 |
| ... | ... | ... | ... | ... |
| 1137 | Proteomics | NADH16 | maximum | 47.052604 |
| 1138 | Proteomics | NADTRHD | maximum | 0.202139 |
| 1139 | Proteomics | NH4t | maximum | 2.957808 |
| 1140 | Proteomics | O2t | maximum | 27.427014 |
| 1141 | Proteomics | PDH | maximum | 20.196188 |

1142 rows × 4 columns

In [21]:

```
fig = px.bar(
    df_plot_b[df_plot_b.Reaction.isin(reacs_to_plot)],
    x="Reaction",
    y="Flux",
    color="method",
    labels="Sense",
    barmode="group",
    template="plotly_white",
    width=800,
    height=600,
)
fig.update_layout(
    legend=dict(
        x=0.03,
        y=1.0,
        traceorder="normal",
        font=dict(family="sans-serif", size=16, color="black"),
    )
)
fig.update_xaxes(title_font={"size": 20}, tickfont=dict(size=16))
fig.update_yaxes(title_font={"size": 20}, tickfont=dict(size=14))
fig
```

In [24]:

```
fig.write_image("fva_comp_reacs.pdf")
fig.write_image("fva_comp_reacs.svg")
```

In [23]:

```
[reac.id for reac in ec_model.reactions if reac.lower_bound == 0]
```

Out[23]:

```
['PFK',
 'PFL',
 'PGL',
 'PPC',
 'PPCK',
 'PPS',
 'AKGDH',
 'PYK',
 'BIOMASS_Ecoli_core_w_GAM',
 'CS',
 'SUCCt2_2',
 'CYTBD',
 'SUCCt3',
 'SUCDi',
 'THD2',
 'EX_ac_e',
 'EX_acald_e',
 'EX_akg_e',
 'EX_etoh_e',
 'EX_for_e',
 'EX_fru_e',
 'EX_fum_e',
 'EX_gln__L_e',
 'EX_glu__L_e',
 'EX_lac__D_e',
 'EX_mal__L_e',
 'EX_pyr_e',
 'EX_succ_e',
 'FBP',
 'FORt2',
 'FRD7',
 'FRUpts2',
 'FUMt2_2',
 'GLCpts',
 'GLNS',
 'GLNabc',
 'GLUN',
 'GLUSy',
 'GND',
 'ICL',
 'MALS',
 'MALt2_2',
 'ME1',
 'ME2',
 'NADH16',
 'NADTRHD',
 'PDH']
```

In [60]:

```
47 / 95 * 100
```

Out[60]:

```
49.473684210526315
```

## Reviewer 2 comment¶

Reviewer 2 asked for significance tests for the distributions.

We are going to run a T-test element wise with all pairwise combinations of methods (Thermo, plain FBA, etc.).

The null hypothesis is that the two vectors arise from the same normal distribution (their mean is equal).

In [63]:

```
from itertools import combinations
```

In [81]:

```
from scipy.stats import ttest_ind
```

In [72]:

```
df_plot = df_plot.reset_index()
```

In [85]:

```
df_plot[df_plot.method == "FBA"].sort_values("reaction")["Flux"]
```

Out[85]:

```
5      20.000000
10     20.000000
11     20.000000
13     20.000000
14     20.000000
         ...    
38     20.154536
39    333.220000
40     20.154536
41     20.466373
42     20.000000
Name: Flux, Length: 95, dtype: float64
```

In [88]:

```
{
    method: df_plot[df_plot.method == method].shape
    for method in pd.unique(df_plot.method)
}
```

Out[88]:

```
{'FBA': (95, 3),
 'Thermo': (95, 3),
 'Thermo + Metabolomics': (95, 3),
 'Thermo + Proteomics': (95, 3),
 'Pool constraint': (96, 3),
 'Proteomics': (95, 3)}
```

We have to remove the pool reaction since they have to have the same lenght to perform the t-test.

In [90]:

```
df_comp = df_plot[~df_plot.reaction.str.startswith("prot")]
```

In [100]:

```
t_results = {
    (method_a, method_b): ttest_ind(
        df_comp[df_comp.method == method_a].sort_values("reaction")["Flux"],
        df_comp[df_comp.method == method_b].sort_values("reaction")["Flux"],
    )
    for method_a, method_b in combinations(pd.unique(df_plot.method), 2)
}
```

In [120]:

```
heat_map = pd.DataFrame({"method1": [k[0] for k in t_results], "method2": [k[1] for k in t_results], "pvalue": [v.pvalue for v in t_results.values()]}).pivot(index="method1", columns="method2", values="pvalue")
```

In [121]:

```
heat_map
```

Out[121]:

| method2 | Pool constraint | Proteomics | Thermo | Thermo + Metabolomics | Thermo + Proteomics |
| --- | --- | --- | --- | --- | --- |
| method1 |  |  |  |  |  |
| FBA | 0.792169 | 0.015763 | 0.608079 | 0.608079 | 0.086291 |
| Pool constraint | NaN | 0.032253 | NaN | NaN | NaN |
| Thermo | 0.802054 | 0.061708 | NaN | 1.000000 | 0.236120 |
| Thermo + Metabolomics | 0.802054 | 0.061708 | NaN | NaN | 0.236120 |
| Thermo + Proteomics | 0.147727 | 0.498918 | NaN | NaN | NaN |

Make symmetric.

In [124]:

```
heat_map.loc["Thermo + Metabolomics", "Thermo"] = heat_map.loc["Thermo", "Thermo + Metabolomics"]
heat_map.loc["Pool constraint", "Thermo"] = heat_map.loc["Thermo", "Pool constraint"]
heat_map.loc["Pool constraint", "Thermo + Metabolomics"] = heat_map.loc["Thermo + Metabolomics", "Pool constraint"]
heat_map.loc["Thermo + Proteomics", "Thermo"] = heat_map.loc["Thermo", "Thermo + Proteomics"]
heat_map.loc["Thermo + Proteomics", "Thermo + Metabolomics"] = heat_map.loc["Thermo + Metabolomics", "Thermo + Proteomics"]
```

Show in order.

In [127]:

```
pip install tabulate
```

```
Collecting tabulate
  Using cached tabulate-0.9.0-py3-none-any.whl (35 kB)
Installing collected packages: tabulate
Successfully installed tabulate-0.9.0

[notice] A new release of pip is available: 23.1.2 -> 23.2.1
[notice] To update, run: /home/georg/.virtualenvs/gecko-cplex/bin/python -m pip install --upgrade pip
Note: you may need to restart the kernel to use updated packages.
```

In [129]:

```
heat_map
```

Out[129]:

| method2 | Pool constraint | Proteomics | Thermo | Thermo + Metabolomics | Thermo + Proteomics |
| --- | --- | --- | --- | --- | --- |
| method1 |  |  |  |  |  |
| FBA | 0.792169 | 0.015763 | 0.608079 | 0.608079 | 0.086291 |
| Pool constraint | NaN | 0.032253 | 0.802054 | 0.802054 | NaN |
| Thermo | 0.802054 | 0.061708 | NaN | 1.000000 | 0.236120 |
| Thermo + Metabolomics | 0.802054 | 0.061708 | 1.000000 | NaN | 0.236120 |
| Thermo + Proteomics | 0.147727 | 0.498918 | 0.236120 | 0.236120 | NaN |

In [128]:

```
heat_map[["Proteomics", "Pool constraint", "Thermo", "Thermo + Metabolomics", "Thermo + Proteomics"]].to_markdown()
```

Out[128]:

```
'| method1               |   Proteomics |   Pool constraint |     Thermo |   Thermo + Metabolomics |   Thermo + Proteomics |\n|:----------------------|-------------:|------------------:|-----------:|------------------------:|----------------------:|\n| FBA                   |    0.0157626 |          0.792169 |   0.608079 |                0.608079 |             0.0862913 |\n| Pool constraint       |    0.0322534 |        nan        |   0.802054 |                0.802054 |           nan         |\n| Thermo                |    0.0617078 |          0.802054 | nan        |                1        |             0.23612   |\n| Thermo + Metabolomics |    0.0617078 |          0.802054 |   1        |              nan        |             0.23612   |\n| Thermo + Proteomics   |    0.498918  |          0.147727 |   0.23612  |                0.23612  |           nan         |'
```
